# Offshore movement by the portunid crab *Scylla serrata* (crustacea: decapoda): new insights into the behaviour and ecology of females migrating to spawn from micro pop-up satellite archival tags

**DOI:** 10.1186/s40462-026-00636-y

**Published:** 2026-03-18

**Authors:** Nicholas J. Stratford, Samuel M. Seghers, Nicole Flint, Julie B. Robins

**Affiliations:** 1 Department of Primary Industries, Northern Fisheries Facility, Cairns, Queensland 4870 Australia; 2 North Rockhampton QLD, Central Queensland University, Rockhampton, 4701 Australia; 3 Department of Primary Industries, Ecosciences Precinct, Brisbane, Queensland 4102 Australia

**Keywords:** microPAT, Satellite tracking, Spawning migration, Great Barrier Reef, Australian east coast, Portunidae, Gulf of Carpentaria, Movement behaviour

## Abstract

**Background:**

Movement to spawn in offshore waters provides numerous benefits to inshore marine fish and crustacean species, but there is often limited empirical data on the behaviour and ecology of this key aspect of their life history. This is the case for Portunid mud crabs of the genus *Scylla*, which have swimming abilities and are widely distributed throughout the Indo-West Pacific.

**Methods:**

Two lines of evidence were used to quantitatively and qualitatively improve the understanding of the spawning migration of *Scylla serrata*, the largest of the four species of mud crab: (i) the novel application of the smallest pop-up satellite archival tag available to twelve females considered to have advanced ovarian development, and (ii) the collation of requested sightings of migrating and/or egg-bearing females. The satellite tags provided empirical data on the movement and behaviour of migrating females, whilst the collated sightings (*n* = 101) provided information on the direction and seasonality of migration. The study occurred between October 2020 and June 2024 in the waters of Queensland, Australia, that included the continental shelves of the Gulf of Carpentaria (approximately 12–18°S, 138–142°E) and the Queensland’s east coast (approximately 11–28°S, 142–153.5°E).

**Results:**

Satellite tag data was obtained from nine individuals, with 30 second interval depth, temperature, and light archived data available from two individuals, including one egg-bearing female. This data indicated three types of behaviour associated with the spawning migration: (i) estuarine benthic behaviour in shallow water (<10 m) where these benthic dwelling crabs remain mostly on the sea floor of tidal estuarine habitats; (ii) active swimming behaviour when the crabs alternated between near surface positioning interspersed with sedentary behaviour at increasing depth likely indicative of swimming to deeper offshore waters; and (iii) offshore benthic behaviour in deeper waters (>20 m). Over 100 sightings of spawning females provided a broadscale insight into their movement and possible offshore destinations.

**Conclusions:**

Results indicated the offshore spawning migration of giant mud crabs is variable, depending on local bathymetric and oceanographic conditions, which has consequences for larval distribution and the genetic and demographic connectivity of this species. Pop-up satellite archival tags can provide novel insights into the spawning migration of brachyuran crabs, providing additional information to inform fisheries management.

**Supplementary information:**

The online version contains supplementary material available at 10.1186/s40462-026-00636-y.

## Background

Movement patterns determine the spatial, demographic and genetic structure of marine species, which often display multi-phase ontogeny [1]. Many inshore marine fish and decapod crustaceans display a tri-phasic life cycle that includes planktonic eggs and larvae, juvenile use of shallow-water inshore habitats and ontogenetic shifts in habitat and resource use by adults [1]. A tri-phasic life cycle includes the movement (often passive) of planktonic larvae from spawning habitats and movement of adults to them. The spawning migration of adults is different from routine movements associated with feeding activities occurring within a species-specific home-range. The primary purpose of spawning migration by adults is to reach areas with suitable conditions that increase the chances of larval survival [2, 3]. Many marine species exhibit spawning migration [4], with numerous decapod crustaceans displaying spawning migration toward offshore areas [2, 5].

As a decapod crustacean and portunid with good swimming ability, mud crabs of the genus *Scylla* migrate to spawn [6–10]. There are four species (*serrata, olivacea, tranquebarica, paramamosain*) widely distributed throughout the Indo-West Pacific, many of which are widely cultivated in aquaculture operations [11] and some species (e.g., *S. serrata*) incurring heavy fishing pressure in wild-capture fisheries (e.g. South Africa, Red Sea, Asia, Australia, Fiji, southern Japan). They have a planktonic larval stage that provides broadscale genetic connectivity [12–14]. Juveniles and adults occur in shallow-water (<10 m), mangrove-associated habitats of estuaries and coastal areas. Females are inseminated during moulting to their mature instar, retaining spermatophores in their spermatheca for internal fertilisation during extrusion of eggs. The egg-mass is incubated on their abdominal flap for between 9 and 14 days, dependent on temperature and salinity [15, 16]. Of the few studies on the spawning migration of *Scylla* [6, 9, 10] considerable variability is reported in the details e.g. distance offshore, depth, seasonality.

Regional variation in their spawning migration is speculated to be related to oceanographic conditions and/or geographic features [17] and is congruent with oceanographic particle simulations of effective larval advection patterns [18, 19]. However, there is limited empirical data on the behaviour and ecology of the spawning migrations of female mud crabs. It is currently unclear what determines the migration pathway and/or endpoint, why the distance travelled offshore varies, and whether, how or what proportion of females return to the coastline after spawning [6, 7, 9, 10].

The current study used two lines of evidence to gain empirical and theoretical insights into the behaviour and ecology of the spawning migration of *S. serrata*: (i) the novel application of micro pop-up satellite archival tags (PSATs) to crabs imminent to their spawning migration; and (ii) collated sightings of migrating and egg-bearing females. The water of the continental shelves of northern Australia were used as a case study for this research, providing a large spatial context, diverse and complex continental shelf habitats, covering tropical and sub-tropical waters and a range of seasonal temperature and rainfall conditions – parameters which have been previously suggested as the triggers of the spawning migration [6, 10].

## Methods

### Study locations

**Fig. 1:**
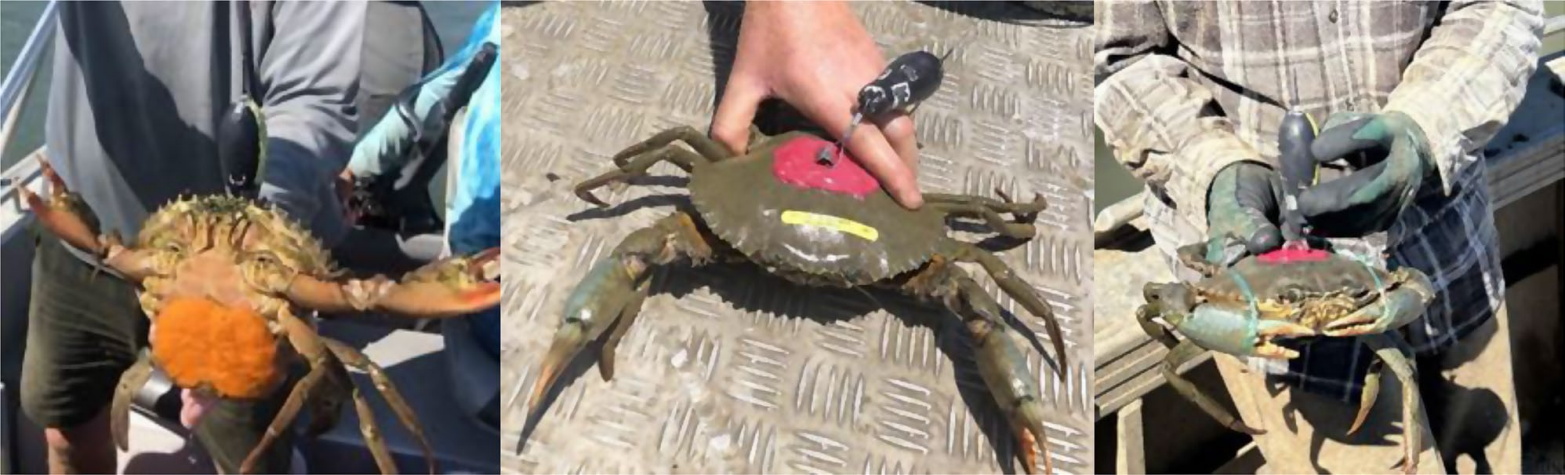
Female *Scylla serrata* showing micro pop-up archival tag (microPAT) attachment method so extrusion of an egg-mass would not be impeded. Examples shown are: (left) crab-253088, an egg-bearing female; (middle) crab-253096; (right) crab-252950. See Table 1 for details

The study occurred in Queensland waters, which include an eastern facing coastline and associated continental shelf open to the Pacific Ocean (approximately 11–28°S, 142–153.5°E), and a western and northern facing coastline open to a semi-enclosed shallow sea and continental shelf that is the Gulf of Carpentaria (approximately 12–18°S, 138–142°E, Fig. 1). These coastlines have extensive areas of mangrove-lined estuarine habitats, supporting significant fisheries for *S. serrata* (approximately 700 tonnes per annum combined, male only harvest [20]), but differ greatly in their oceanography and bathymetric complexity.

The Queensland east coast extends for over 2222 km from Cape York (~10.68°S) to the New South Wales border (~28.17°S). Offshore of estuaries inhabited by *S. serrata* is the Great Barrier Reef, which occurs from Cape York to ~25°S. The Great Barrier Reef provides a complex continental shelf that includes coral reefs (both eastern outer boundary reefs and inner individual reefs), continental islands, and coral sand cays dispersed beside and amongst a deeper ‘lagoon’ area that occurs between the mainland (where most mangrove-lined estuaries are located) and the outer barrier reef. Shallow water (<20 m) occurs in a narrow area generally within 3 km of the coast [21], seaward of which depth increases across the continental shelf, until the 100 m depth contour (considered the eastern boundary). East of the 100 m depth contour is the drop-off to the continental slope. The width of the continental shelf from the mainland coast to the outer reef averages 100 km but ranges from 23 km (at 14°S) to 260 km (at 22.5°S). Within the Great Barrier Reef, currents are a function of semi-diurnal tides and prevailing seasonal winds, which are northwesterly during the monsoon season (~October to April) and southeasterly during the tradewind season (~May to September). Seaward of the outer barrier reefs, the westward flowing South Equatorial Current (and jets) splits when it hits the outer reefs (~17°S [22]), creating complex currents that flow either generally northward (i.e., North Queensland Current and Hiri Gyre) or generally southward (i.e., East Australian Current) [22]. South of the Great Barrier Reef, parts of the Queensland east coast are sheltered from the Pacific Ocean by large, sand barrier islands (K’gari, Bribie, Mulgumpin, North and South Minjerribah). The offshore waters south of Sandy Cape (K’gari, ~24.68°S) are greatly simplified in comparison to the offshore waters of the Great Barrier Reef, generally characterised by fine sandy sediments, a narrow continental shelf, with an eastward continental slope that rapidly increases in depth. Offshore waters south of Sandy Cape are exposed to the dynamics (e.g., temperature, velocity, and direction) of the East Australian Current and its associated eddies.

The Gulf of Carpentaria is a semi-enclosed sea with a coastline extending for approximately 1852 km from Cape York (~10.68°S, 142°E) to Cape Wessel (11°S, 136.75°E), of which 1111 km is within Queensland’s jurisdiction. The coastline has few major headlands and there are extensive, wide (up to 20 km) and shallow (<20 m) coastal flats that are turbid and well-mixed [21]. Offshore, water depths increase to a maximum of 65 m, and are oceanic in quality, being separated from inshore coastal waters by a boundary current [23]. Within the Gulf of Carpentaria, currents are a function of diurnal tides and prevailing seasonal winds, which are northwesterly during the monsoon season and southeasterly during the trade wind season [24, 25]. Although occurring within tropical latitudes (i.e., ~10.68°S to 18°S), inshore coastal waters of the Gulf have larger seasonal variation in temperature than inshore coastal waters of equivalent latitude on the Queensland east coast and Great Barrier Reef [26]. The diurnal tides, and adjacent land features, amplify the seasonal range of water temperatures on the inshore coastal flats and adjacent estuaries.

### Satellite tagging

Acoustic and satellite tags were considered to study the spawning migration as both have been previously used to study crab movement [e.g. 10, 27, 28]. Pop-up satellite archival tags (PSATs) were selected because they had successfully provided location data in previous crab migration studies [27, 28], and they can provide a time-series of temperature and depth data to inform movement behaviour, which acoustic tags do not. Migrating female *S. serrata* are unlikely to spend sufficient time at the surface to allow the use of tags which transmit data in real-time (e.g., SPLASH or SPOT tags). Wildlife Computers microPATs were chosen as they were the smallest pop-up archival satellite tag available (i.e., 95 mm × 33 mm, plus antennae, weighing 46 grams in air).

Upon deployment, microPATs record depth, temperature and light information, with their programmed release from the animal occurring by a burn-pin, detaching the tag from its tether to the animal. Once released, the microPAT, floats to the surface and a wet-dry sensor notifies the tag that it has surfaced, after which the tag transmits data to the Argos satellite network. MicroPATs provide a pop-up location as well as depth, temperature and light level readings which can be used to generate light-based estimates of geolocation [29]. If the tags are physically recovered, the complete archived data series can be downloaded, otherwise ‘packaged’ data consisting of daily minimum (min) and maximum (max) depths, temperatures and light levels over six-hourly intervals are transmitted to the Argos satellite network whilst the microPAT has capacity (i.e., battery life and clear line of transmission). We set the microPATs to record depth (0.5 m resolution), temperature and light data every 30 seconds, with the release-pin set to burn at 30, 45, or 60 days depending on the time of year and condition of the crab (Table 1). The egg-bearing female had tag-release set at 30 days, whilst females without eggs had tag-release set to 45 days (April) or 60 days (October) to enable further ovary development prior to their spawning migration. Transmission repetition rate was set to 60 seconds, with pop-up locations based on transmissions to Argos satellite network confirmed by messages detected by two, preferably three satellites.

**Table 1:** Details of female *Scylla serrata* deployed with microPAT satellite tags in Queensland waters – East Coast (EC) and Gulf of Carpentaria (GoC). No data received = never heard from the tag. n/a = not applicable

Location	PTT ID	CW mm	Release date	Release lat/long	Pop-up date	Pop-up lat/long^b^	Days at liberty, early release, reason if known	Pop-up depth (m)	Distance release to pop-up location (straight line km)
Karumba, GoC	253,092	166	04/10/23	−17.46518 140.82823	na	na	no data received	n/a	n/a
	253,090	160	05/10/23	−17.46518 140.82823	04/12/23	−16.61947 140.43152	60 days	19	103
	253,091	169	05/10/23	−17.46518 140.82823	04/12/23	−16.57403 140.50987	60 days	21	106
	253,093	178	05/10/23	−17.46703 140.82058	15/11/23	−17.00323 140.95843	41 days, early release reason unknown	19	54
	253,096	167	05/10/23	−17.46703 140.82058	04/12/23	−16.95478 140.38788	60 days	19	73
Missionary Bay, EC	253,088^a^	150	17/10/23	−18.20757 146.22158	16/11/23	−18.157483 146.29133	30 days	18	11
	253,089^a^	157	17/10/23	−18.26597 146.23190	na	−17.8925 146.09923	18 days, early release, predated	15	38
	253,094	147	17/10/23	−18.20755 146.22158	na	na	no data received	n/a	n/a
	253,095	168	17/10/23	−18.20757 146.22158	16/12/23	−19.1795 149.51493	60 days	320	363
	253,097	156	17/10/23	−18.27295 146.19705	na	n/a	no data received	n/a	n/a
The Narrows, EC	262,950	163	11/04/24	−23.55920 150.95838	04/05/24	−20.20893 150.04338	23 days, early release, WetDry function triggered	69	387
	262,949	166	11/04/24	−23.55920 150.95838	17/04/24	−23.5155 150.81192	6 days, early release, depth function triggered	<1 m	21

CW = carapace width; ^a^ full archival data obtained forrecovered tags; ^b^ Argos Location QualityClass-3, ≥4 messages per satellite pass, estimated location error < 250 m

The microPATs were coated with antifouling paint (International Interprotect High Performance Epoxy Primer as an undercoat and International Ultra 2 High Strength Antifouling as a topcoat [30]) as per Wildlife Computers recommendation. Harness attachment methods utilised in previous brachyuran PAT studies [27] were inappropriate as obstruction to the opening of the abdominal flap would prevent egg extrusion and subsequent collection and attachment of the eggs to the crabs abdominal pleopods. We developed a system whereby the microPAT was attached to the crab using flexible stainless-steel wire (Grade 316, 7x7, 0.81 mm in diameter) looped and crimped to a stainless-steel ‘saddle’ that was glued with an adhesive to the dorsal carapace (Fig. 1). The carapace was wiped clean then abraded to enhance bond strength and a generous bead of epoxy was applied to adhere the stainless-steel saddle to the dorsal carapace of the crab. We opted for an epoxy footprint of approximately 5 cm by 2 cm to provide strong adhesion without interfering with the animals’ natural behaviours. Prior to deployment on wild individuals, tank trials were conducted to explore attachment options, adhesive types and buoyancy effects of the microPAT on female crabs. We tested several adhesives including multiple brands of 5-minute epoxy. Iccons Pure Epoxy [31] was selected for field application as it created the strongest bond, did not produce significant heat when curing and did not soften or weaken after long periods of submersion in sea water. Tagged crabs were held and observed in tanks for 7 days, during which the natural movements and buoyancy of the individuals did not appear to be hindered by the tethered microPAT, consistent with Nault et al. [32].

Locations for microPAT application to female *S. serrata* were based on: (i) the logistics of collecting females with advanced ovary development (described below), (ii) estuarine habitat complexity to minimise the risk of tag entanglement, (iii) relevance to high-catch fishery areas, and (iv) limited information (published or anecdotal) on spawning movement (i.e., knowledge gap). Egg-bearing female sighting data [6] and larval modelling [18, 19] led to the deployment of 12 microPATs across three locations - Missionary Bay and The Narrows, both on the Queensland east coast, and Karumba in the Gulf of Carpentaria (Table 1).

**Fig. 2:**
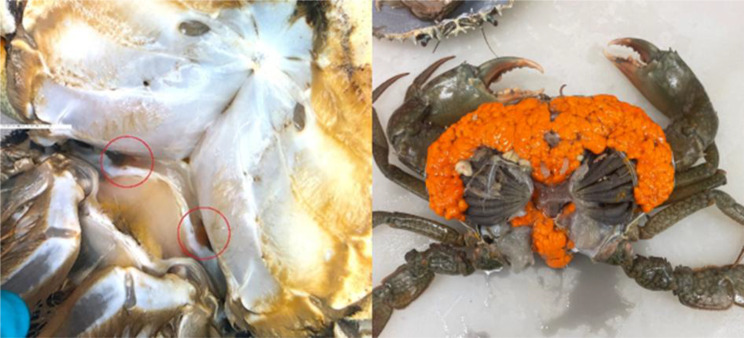
Externally visible indicators of late-stage V (i.e., fully mature) ovary development in female *Scylla serrata*): (**a**) orange crescents (inside red circles) visible through the transparent membrane between the junction of the first abdominal segment and the ventral carapace (thoracic sternites), and (**b**) dissected late-stage V female showing full mature ovary (orange tissue)

The microPATs were deployed in the austral spring and autumn when female *S. serrata* are thought to have peak spawning [6, 10, 17]. Females were collected with the assistance of local commercial fishers from baited 30 ply polyethylene mesh covered crab pots, which had been set in the intertidal zone. Mature females with limbs intact and showing advanced ovary development were selected for tagging. Females in advanced stage-V ovary development (i.e., tertiary vitellogenesis [33]) were assessed by visual examination of the transparent membrane between the junction of the first abdominal segment and the ventral carapace (thoracic sternites). The ovary of advanced stage-V females fills the body cavity to such a degree that the ovary (usually orange in colour) extends into the abdominal segment of the crab (i.e., tail flap), resulting in orange crescents being visible externally (Fig. 2). Observation of orange ovary material in the abdominal flap of female mud crabs is used in aquaculture to identify when spawning is imminent (i.e., expected to occur within two to three weeks [34] and was verified by dissection in concurrent research (Robins unpublished)). Following tag application, crabs were held in individual containers, covered with hessian and kept cool and damp until the epoxy was set (90–120 minutes, temperature dependent). Individuals were released as close as feasible to their capture location.

Location processing from the deployed microPATs using GPE3 provided in the Wildlife Computer Data Portal was attempted for all individuals with data transmitted to Argo satellite network or where the microPAT was retrieved and full data downloaded. Modelling of the movement track of the microPAT tagged crab had variable success and overall was not more robust or that much more informative than the straight-line track presented in the Results. Best fits (based on maximum likelihood ‘score’ (0 to 100), and plausibility of movement) of the geolocation processing are provided in the Supplementary Material for 6 of the microPAT tagged crabs. Three of the PSAT data sets were unable to have a track estimated by GPE3 processing due to lack of model convergence.

### Collated sightings of migrating and egg-bearing females

**Fig. 3:**
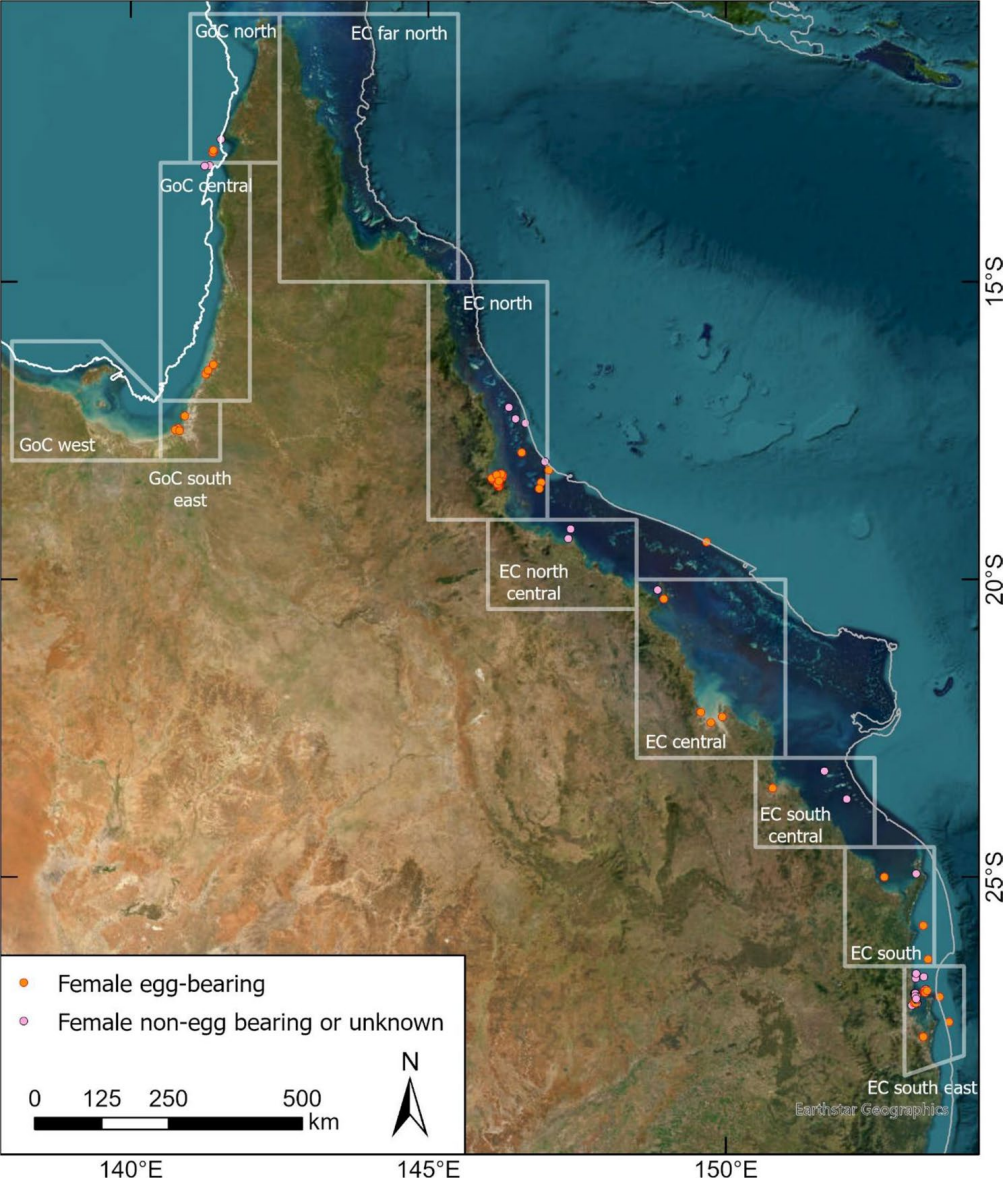
Locality map of study and spatial distribution of female *Scylla serrata* considered to be undertaking spawning migration in Queensland waters. Egg-bearing status was confirmed by photograph. White boxes illustrate fishery regions (see Supplementary Material Table S1). GoC = Gulf of Carpentaria. EC = Queensland east coast

Sightings of female *S. serrata* ‘offshore’ and egg-bearing females were collated from social media posts (Instagram, Facebook), and directly from commercial and recreational fishers following public announcements requesting the reporting of such sightings (see Supplementary Material). Collated information included the source of information, date of sighting, location (latitude and longitude, or bearing and distance from a landmark), water depth, photographic evidence (to confirm species, whether egg-bearing, colour of egg mass to infer development stage), plus other relevant comments (e.g. sighted at surface, type of fishing activity – pot or trawl caught). Only sightings confirmed as *S. serrata* with precise location information were utilised. Photographs of the egg-mass were visually assessed (colour, larval development) to roughly estimate development (i.e., days post extrusion) guided by expert opinion [15, 35, 36]. Where possible, perpendicular distance to the nearest mainland shoreline was calculated. The sightings were aggregated by region - seven for the Queensland east coast and three for the Gulf of Carpentaria reflecting geography and climate patterns, to assist in understanding potential regional patterns in spawning migrations (Fig. 3, regional boundaries provided in the Supplementary Material). Seasonality of spawning migration was investigated by assessing the monthly frequency of sightings.

## Assumptions and limitations

### Sample size

Twelve microPATs were deployed with informative data retrieved for nine (Table 1). Thus, the sample size is small and a limitation that should be considered when interpreting results. However, the female *S. serrata* were tagged and released in three different locations, and two different times of year providing a degree of representativeness. The number of microPATs deployed was restricted by funding and uncertainty as to whether microPATs could return any data on the spawning migration of this species. The microPAT derived data (reported in the Results) provides water depth and temperature data for individuals and is suggestive of behaviour, and whilst variable has commonalities. We suggest our interpretation is not unreasonable, as it concurs with the cited published literature (see Results and Discussion) and is likely applicable to the wider population.

### microPAT weight relative to crab weight

Crabs fitted with microPATs ranged in size from 147 to 178 mm CW (Table 1), with an estimated weight between 605 and 980 grams respectively (Robins unpublished). As microPATs weight 46 grams out of water, the tag-weight to animal-weight ranged between 7.6% and 4.7% and was greater than the suggested 2% rule for tags applied to fish [37], although other studies where PSATs were applied to crabs had fish-tag to animal-weight values between 10% and 1.9% [27, 28]. PSATs have been reported to not change the crabs natural movements or buoyancy [32] – consistent with our observations during the 7 day tank trials, suggesting that the 2% fish-tag ‘rule’ may be overly-conservative [38].

### Collated sightings

The collated sightings are opportunistic, a limitation that should be considered when interpreting the results. However, given the almost limited empirical data on the spawning migration of *S. serrata*, these sightings provide useful insight into the regional seasonality, migration directions and potential end points of the spawning migration, particularly when combined with the microPAT results. The extensive distribution and abundance of female *S. serrata* in Queensland waters, noting that harvest is prohibited in this jurisdiction, and the intensity of commercial and recreational fishing effort, would imply that migrating females should be sighted (even if at low frequencies) in all regions, with the chance of sightings increasing in proportion to the level of human activity in a region.

## Results

### Satellite tag performance

Of the 12 microPATs deployed, nine (75%) transmitted data to the Argos satellite network, of which eight had informative data (Table 1). Three microPATs (25%) were never heard from and no data was received by the Argos satellite network. Five of the tags that transmitted data (55%) remained attached for the programmed duration, while four (45%) released early, one likely due predation but the others for unknown reasons (Table 1 and discussed further below). Two microPATs were retrieved, one of which also transmitted to the Argos satellite network (i.e., ID 253,088), providing archived data collected at 30 second intervals for 30 days post-release. This enabled comparison between the 6 hourly summary data transmitted to the Argos satellite network and the microPAT archived depth, temperature and light data. As presented below, the 6 hourly summary data captured key changes in depth and was consistent with that recorded in the 30 second interval archived data, giving confidence that the summary data provides credible information on the spawning migration. To illustrate inferred behaviour, examples of depth and light are presented below, with the data (including temperature) for each microPAT plotted in the Supplementary Material.

### Movement, depth and inferred behaviour

**Fig. 4:**
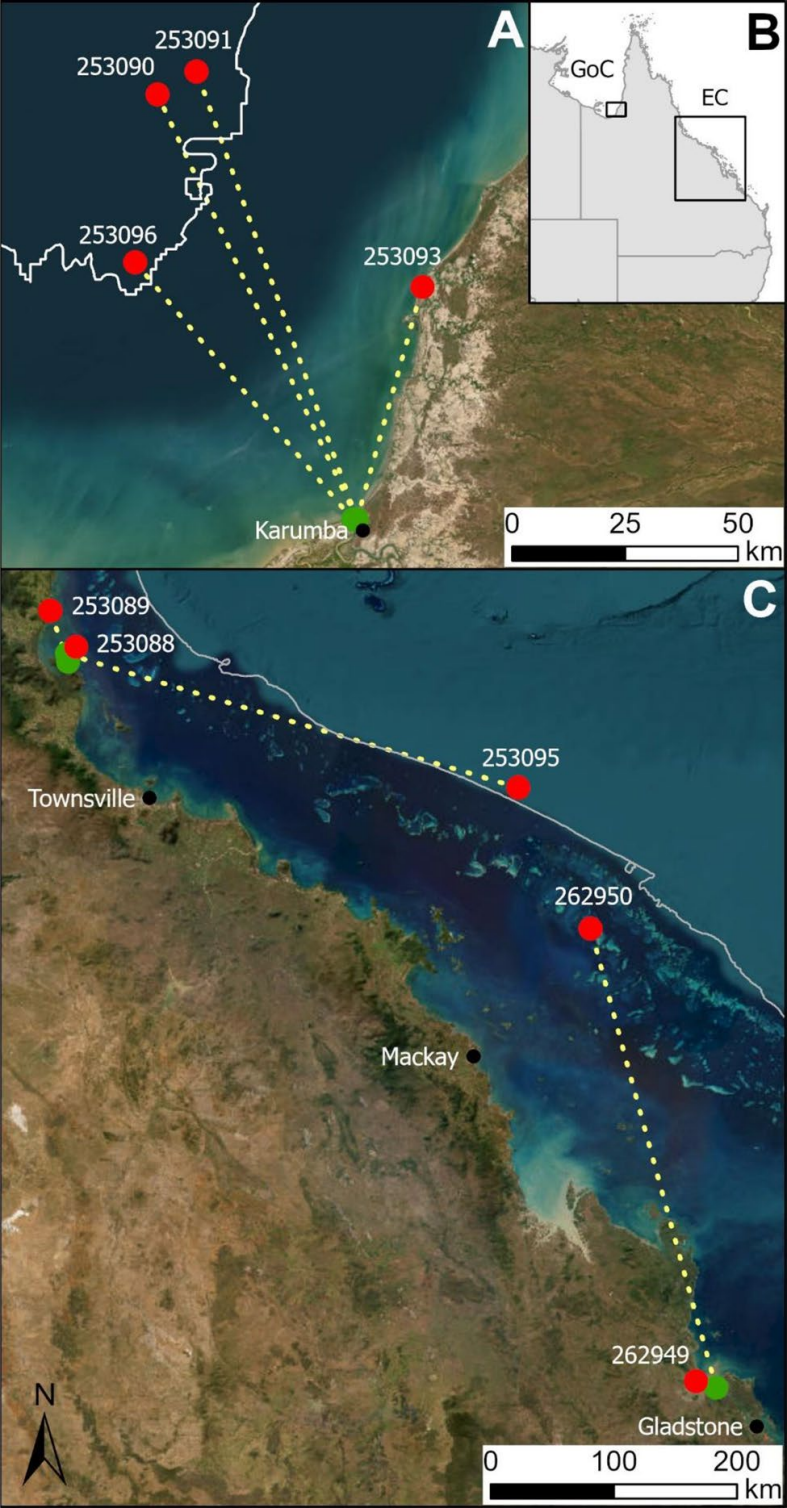
Release (green markers) and pop-up location (red markers) of microPATs attached to female *Scylla serrata* for tags where data was transmitted to the Argos satellite network or the tag retrieved. See Table 1 for details. Yellow dashed line is the straight-line path of least distance. (**A**) south-east Gulf of Carpentaria (GoC) with 20 m depth contour indicated (white line). (**B**) Locality map. (**C**) Queensland east coast (EC) with the 100 m contour line indicated (grey line)

**Fig. 5:**
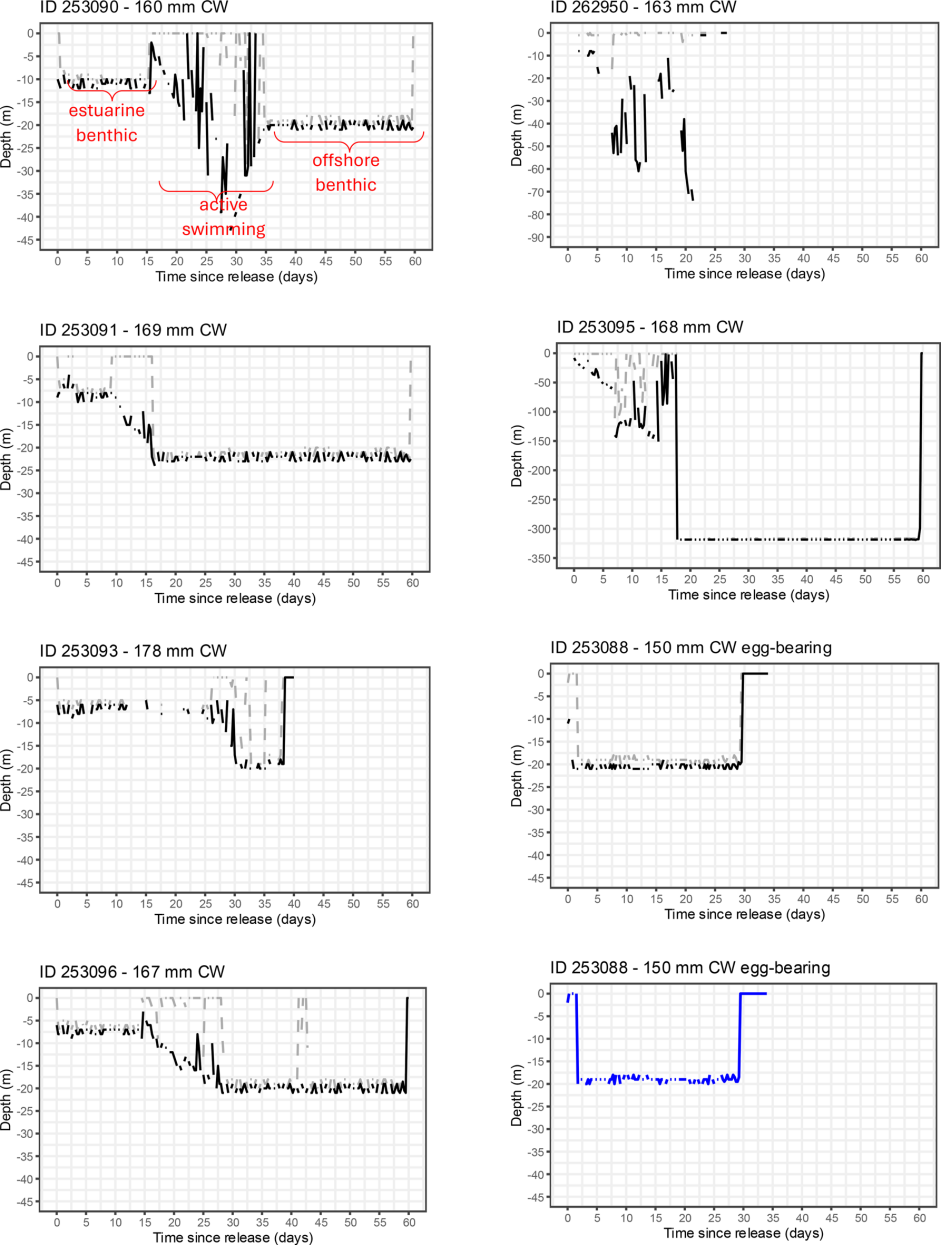
Depth profiles of data derived from microPATs attached to female *Scylla serrata* released in the south-east Gulf of Carpentaria (left-hand plots) and Queensland east coast (right-hand plots). See Table 1 for details of each crab released. CW = carapace width. Six-hourly minimum and maximum depth data transmitted to the Argos satellite network indicated in black. Recorded depth at 30 second intervals archived on the retrieved microPAT indicated in blue. Behaviour phases (estuarine benthic, active swimming, offshore benthic – see text for details) indicated in red, exemplified in the depth profile for crab ID 253,090

Data was available for four of the five females tagged in the Karumba region (south-east Gulf of Carpentaria) and showed movement away from their estuarine release location to deeper water (Fig. 4). The straight-line distance between the release and pop-up location ranged between 54 and 106 km north or north-west of the release location (Table 1, Fig. 4). The 6 hourly summary data transmitted to the Argos satellite network indicated similar patterns in the minimum (min) and maximum (max) depth experienced by these individuals (Fig. 5). Immediately post-release (“estuarine benthic”), the min and max depth values remained approximately the same (usually ≤ 10 m) indicating the benthic behaviour of the tagged crabs on the sea floor, likely in inshore or estuarine waters given the bathymetry of the south-east Gulf of Carpentaria. The duration of the estuarine benthic phase ranged from 9 days (crab-253091) to 26 days (crab-253093). After that (“active swimming”), min depth values were near 0 m (indicating crab at surface) interspersed with max depth values increasing to 20–40 m (Fig. 5), indicating the tagged crabs were at the surface (likely swimming, a behaviour commonly reported in the sightings) interspersed by the crabs at or near the sea floor (likely sedentary or walking, behaviours common in estuarine locations). Lastly, (“offshore benthic”) min and max depth values were approximately the same but at greater depth (i.e., approximately 20–25 m), indicating tagged crabs remained at or near the sea floor, at considerable distance from shore, given the bathymetry of the south-east Gulf of Carpentaria. Tag release, via programmed pin-burn at the expected time and date, was visible in the 6 hourly data transmitted to the Argos satellite network, with min and max depth values abruptly changing to approximately 0 m (Fig. 5). Further evidence that similarity in the min and max depth values was indicative of benthic behaviour, is that variation in the min and max depth values follows the tide cycle (which is diurnal) and amplitude of the Karumba region.

Temperature-at-depth indicated that all the tagged females encountered water temperatures that were initially 26–28 °C, which increased over the duration of tag-deployment to approximately 30 °C (see Supplementary Material). At water temperatures of 29–30 °C, egg incubation in *S. serrata* is estimated to take approximately 11 days [39]. The microPATs provided no information as to whether the tagged females extruded an egg-mass, incubated the egg-mass, or what subsequent activity may have occurred (e.g., extrude another batch at an offshore location). However, depth profiles of the female *S. serrata* released at Karumba provided no evidence to support that migrating females returned to shallow inshore or estuarine waters post-spawn.

The Queensland east coast has greater diversity and complexity in its ‘offshore’ bathymetry and oceanography compared to the Gulf of Carpentaria. Thus, we expected greater variability in the spawning migration of east coast females, in terms of distance moved offshore, depth of water traversed, and direction of movement (i.e., north, south or east). Data was available for four of the seven tagged females released in two locations along the Queensland east coast. The straight-line distance between the release and pop-up location of the microPATs ranged between 11 and 387 km, with movement in northern, easterly and southern directions, noting that two tags popped up near or beyond the outer Great Barrier Reef (i.e., 100 m contour, Fig. 4). Depths recorded by tagged crabs on the east coast were more variable than those recorded for the Gulf of Carpentaria, with max depth values ranging from approximately 25 m (crab-253088) to 320 m (crab-253095, Fig. 5).

The behaviour of females on the east coast was consistent to that observed for females in the Gulf of Carpentaria in that the crabs were initially estuarine benthic, then actively swimming, with two profiles showing subsequent offshore benthic behaviour at deeper depths (Fig. 5). Crab-253095 ‘settled’ at 320 m water depth, likely located beyond Great Barrier Reef continental shelf waters given its pop-up location (Table 1, Fig. 4). Recorded water temperature at 320 m (derived from the Series Range file) was 13 °C (see Supplementary Material) and unlikely to enable successful egg incubation. We surmise that at some point during the tagged period, crab-253095 had died. Detailed inference on the possible movement of this crab is provided in the Supplementary Material. The other east coast crab to show offshore benthic behaviour subsequent to active swimming behaviour, was the egg-bearing crab-253088, which settled in water approximately 20 m deep, likely located within Great Barrier Reef continental shelf given its pop-off location (Table 1, Fig. 4). Recorded water temperatures of 26–27 °C (see Supplementary Material) were almost optimal for egg-development, with incubation expected to take 10–15 days [34, 39].

**Fig. 6:**
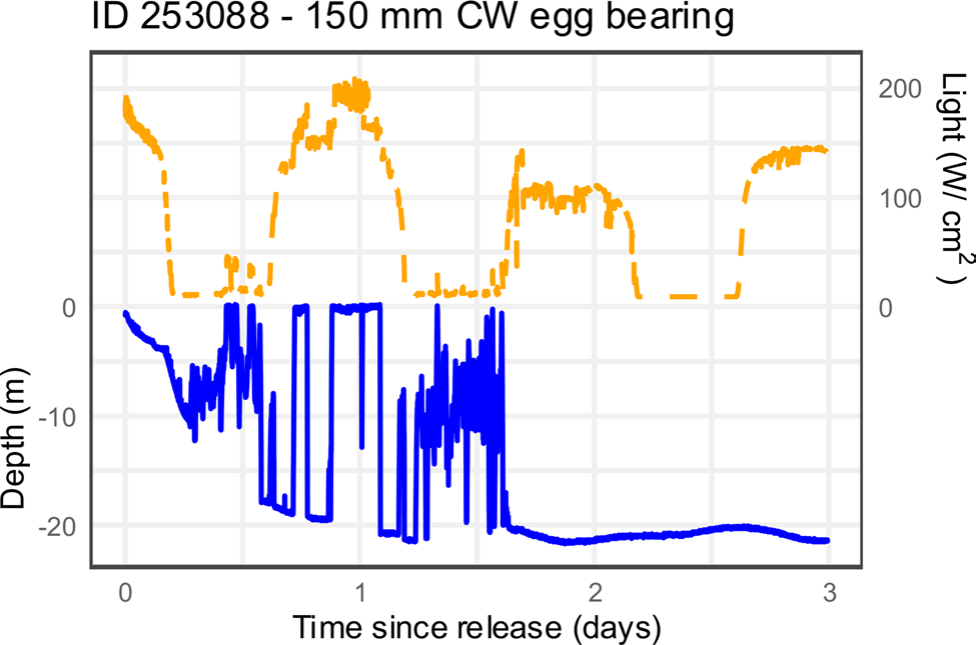
Depth and light data (at 30-second intervals) for the first 3 days post-release from retrieved microPAT 253,088 that was applied to an egg-bearing female *Scylla serrata*, illustrating behaviour patterns of swimming at surface (depth ~ 0 m) interspersed with short-term variable or stable depth, and then long-term stable depth

**Fig. 7:**
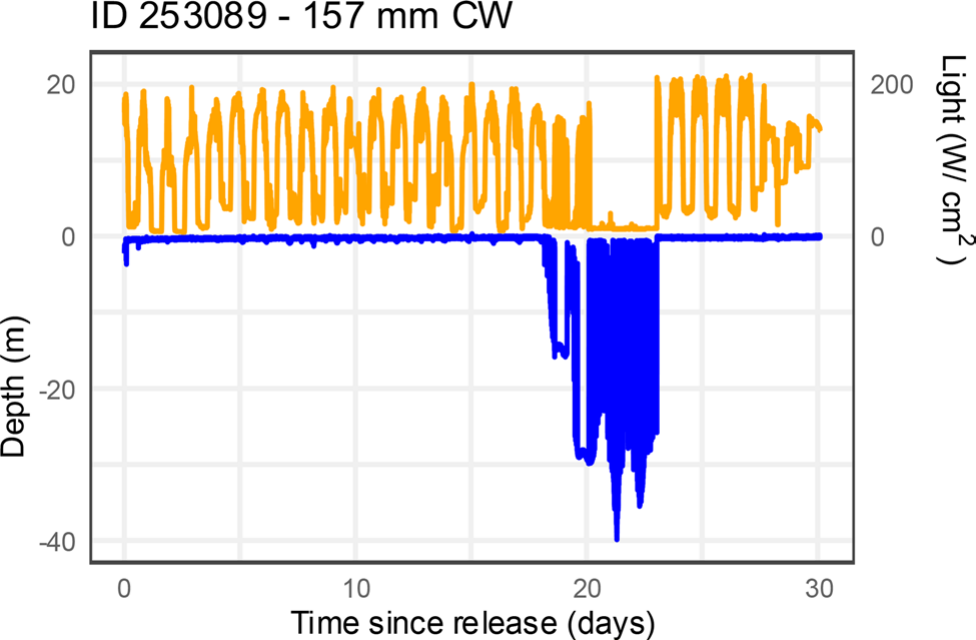
Depth and light data (at 30-second intervals) for 30 days post-release from retrieved microPAT 253,089 that was applied to a female *Scylla serrata*. likely predated, as indicated by light levels below 20 W/cm^2^ between 20 and 23 days post-release

MicroPAT 253,088 was retrieved, providing a 30 day profile of depth recorded at 30 second intervals (Fig. 5), with detailed depth and light data for the first 3 days post-release provided in Fig. 6. The archived data shows details of the “active swimming” behaviour and then subsequent “offshore benthic” behaviour. MicroPAT 253,089 was also retrieved, being recovered before its programmed release and data transmission. This tag was recovered from a beach 38 km northwest of its release location (Table 1, Fig. 4), showing evidence of predation i.e., missing its antennae and covered in teeth marks. The archived data indicated that this crab had remained in shallow water (<2 m) for about 18 days post-release (i.e., behaviour consistent with “estuarine benthic”), before the data indicated offshore movement (i.e., “active swimming” with depths increasing to 26–28 m interspersed with shallower depths, Fig. 7). At approximately 20 days post-release, the light data, which had up to this point tracked the sunrise and sunset cycle, showed a 72 hour period of darkness (i.e., light level below 20 W cm^−2^) along with stable temperature (in contrast to preceding variable temperature, see Supplementary Material) and extremely variable depth – taken as evidence of ingestion of the tag (and possibly the crab) by a predator. At approximately 23 days post-release, the light data returned to a diurnal pattern, suggesting the microPAT had passed through the predator and floated for approximately 4 days (stable depth and temperature) before washing ashore (cycling temperature indicative of day/night air temperatures) approximately 28 days post-release.

### Sightings of migrating and egg-bearing females

**Fig. 8:**
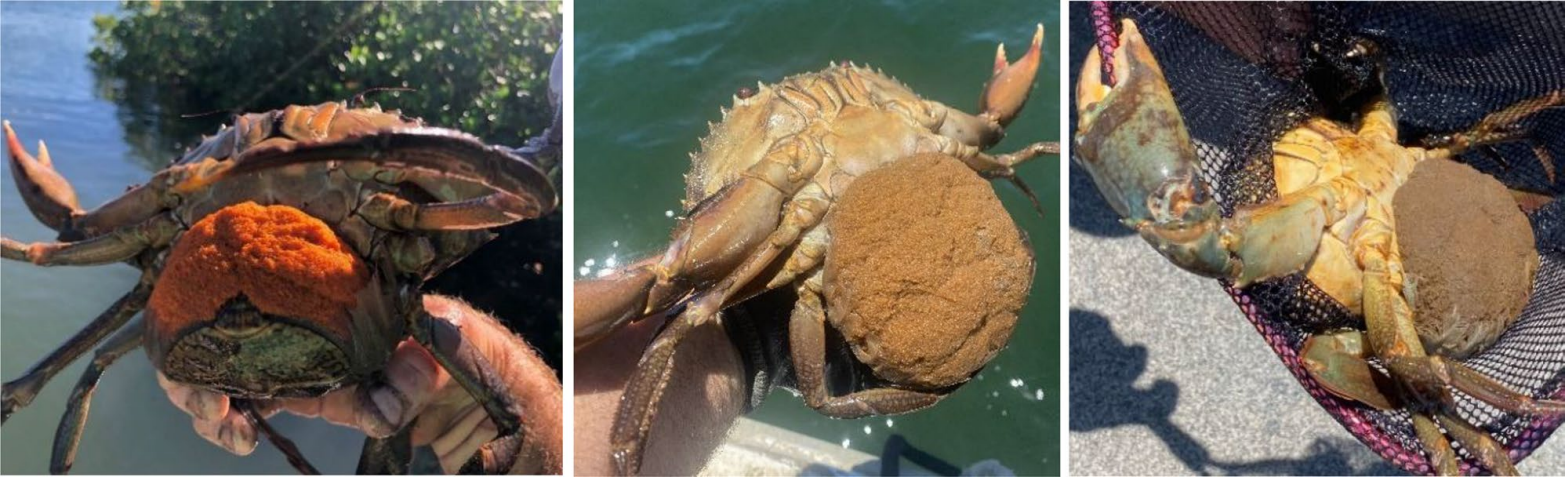
Examples of reported egg-bearing female *Scylla serrata*, showing range of egg-mass development, estimated based on colour as approximately 3, 8 and 11 days post extrusion, images left to right respectively

Between October 2020 and June 2024, we collated 101 reported sightings of female *S. serrata* in offshore locations (i.e., well beyond estuarine habitats) and egg-bearing females in inshore locations (i.e., within estuarine habitats). Most of the egg-bearing females were reported with bright orange egg-mass (*n* = 62), suggesting extrusion was within the past few days (Fig. 4), although multiple brown to dark-brown egg-masses (*n* = 7), signalling late-stage egg development (i.e., approximately 8–11 days post extrusion) were also observed (Fig. 8).

Female *S. serrata* were reported mostly from the southern (*n* = 38) and northern (*n* = 36) regions of the Queensland east coast (Fig. 1, further details in Supplementary Material, Table S2). In the southeast region of the east coast, sightings were most frequent in October and November (*n* ≥ 8), with three or fewer sightings in all other months. Whilst reports included trawl-caught and recreational fisher sightings, most reports were of crabs caught in commercial crab pots targeting *Portunus armatus* (blue swimmer crabs) in nearshore oceanic waters adjacent to a barrier sand island at the northern end of Moreton Bay (i.e., Bribie Island, adjacent to the city of Brisbane). In the north region (Queensland east coast), the highest number of sightings occurred in September (*n* = 8), October (*n* = 6) and November (*n* = 7), with four or fewer sightings in all other months (see Supplementary Material, Table S2). Most reports were of egg-bearing females caught in commercial crab pots targeting legal males in inshore estuarine areas. In the other regions, limited inference can be made on the seasonality of the spawning migration because of low number of reported sightings. However, the four female *S. serrata* reported in October in the north region of the GoC is similar to the seasonality reported by Hill [6]. Females reported from offshore locations included both egg-bearing and non-egg bearing individuals, usually sighted swimming at the surface. Offshore sightings occurred in water depths ranging from 12 to 140 m, and distances of 5–130 km from shore. Females reported from inshore locations were only considered if they were egg-bearing or in atypical locations. Inshore and estuary sightings of egg-bearing females occurred in water depths ranging from <5–18 m, and distances of within estuaries to 9 km from shore.

## Discussion

The combination of pop-up satellite archival tags and collated sightings has provided new and unique quantitative and qualitative information on the spawning migration of female *S. serrata*. The satellite tags ‘tracked’ females providing novel information on behaviour during the spawning migration, direction of movement and locations where egg incubation may be occurring, whilst the sightings provided more broadscale insight into possible migration pathways and seasonality.

### Satellite tagging

Judicious application of satellite tags successfully provided novel data on the spawning movement of *S. serrata,* a brachyuran crab. Key logistics that supported the successful deployment of satellite tags were: (i) targeting females with signs of advanced ovary development (i.e., externally visible orange crescents in the abdominal flap), (ii) secure attachment of the tag to the carapace in a manner unlikely to impede spawning (i.e., harness attached by glue that did not obstruct the opening of the abdominal flap), and (iii) deployment of microPATs in locations where the entanglement risk (in mangrove prop roots or crab pots) was minimised. Collaboration with commercial fishers who are regularly on the water contributed to the success of the research.

Initially, the tags were programmed to release by automated pin-burn and tag pop-up at 60 days post-release for non-egg-bearing females, and 30 days post-release for the egg-bearing female. The premature release capabilities of microPATs were not activated on the crabs released in Karumba or Missionary Bay because this was the first use of microPATs on a crab species that inhabits estuarine intertidal habitats, where during parts of tidal cycle, the crabs may be in very shallow water or no water at all, which would cause premature release of the tag if certain premature release settings were used. Two of the first 10 microPATs deployed popped-up earlier than programmed, and three of the first 10 microPATs did not successfully transmit any data to the Argos satellite network. If premature release capabilities had been activated, some data from these tags might have successfully transmitted rather than no data at all.

MicroPATs deployed on crabs in The Narrows region were deliberately programmed with activated premature release capabilities based on the following conditions: the wet-dry sensor on the tag was more than 25% dry or was shallower than 1 m, or the tag was at a constant depth of ± 4 m for longer than 120 hours. This was beneficial as one microPAT (ID 262,950, Table 1) transmitted data soon after its release from the crab. However, the chosen automated release settings also resulted in premature release of a tag on a female that stayed in shallow estuarine habitat for longer than 120 hours (i.e., ID 262,949). Premature release could have been avoided by setting the ‘first dive below’ condition to a greater depth (e.g., 50 m).

### The spawning migration of female *S. serrata*

Few studies have reported on the spawning migration of *S. serrata* [6, 10] or other *Scylla* species (e.g., *S. olivacea* [9], with limited empirical quantification of the movement behaviour during this key aspect of their life cycle. In general, the spawning migration is towards an offshore destination, noting there is no clear definition of ‘offshore’ other than some undefined distance from “inshore, especially estuaries” [6]. Variability in the offshore migration of *S. serrata* has been previously noted, as egg-bearing females may occur within a few kilometres of mangrove habitats or up to 50 km from shore [9, 17, 40]. The direction and possible end-points of the spawning migration is speculated to be driven by females seeking suitable and/or stable water quality conditions (e.g., temperature and salinity) to maximise larval survival given regionally (or seasonally) variable oceanographic and geographical features [41], with previous studies focused on distance offshore and water depth [6, 10]. Using satellite tagging technology, the current research has provided unique new insights into some of the abiotic parameters influencing the spawning migration (e.g., water depth and temperature) and/or possible endpoints. Given the diversity of locations within the current study, the results provide the groundwork for further research into this elusive life-cycle phase of *S. serrata.* We suggest tags capable of differentiating walking or swimming from sedentary behaviour (e.g., tags with accelerometers) might be useful to discern active migration from sedentary benthic behaviour.

### Region specific comments – Gulf of Carpentaria

The current study is congruent with the results of Hill [6] and fills a knowledge gap in the south-east region of the Gulf of Carpentaria which had limited sampling [6]. In the Gulf of Carpentaria, female *S. serrata* migrate to offshore waters 20–25 m in depth ([6] and results of the current study), which likely provide salinity and temperature water quality characteristics appropriate for egg-incubation and survival of planktonic larvae [42]. We speculate that spawning migration in the Gulf of Carpentaria is to locations that are beyond the turbid inshore waters, where fine sediment is frequently resuspended by wind and tide [43]. In the Gulf of Carpentaria, oceanographic features, such as the coastal boundary current [23] likely moderate larval dispersal [44], resulting in a degree of east-west genetic structuring across in this a semi-enclosed sea [13, 14].

### Region specific comments – Queensland east coast

The current study provides the first quantitative evidence of the movements, direction and behaviours that spawning female *S. serrata* undertake along the Queensland east coast. Compared to other locations, the Queensland east coast has a highly complex continental shelf with a diversity of sea floor sediments (e.g., mud, sand, coral/shell grit) and current regimes. Most sightings of egg-bearing females along the Queensland east coast were from either the south-east or north regions – likely an artefact of the opportunistic nature of this data collation. In the south-east region, sightings were most common in October in oceanic areas slightly offshore of the Moreton Bay barrier islands. In the north region, sightings were most common between September and November, being reported in estuarine areas adjacent to Hinchinbrook Island. Many of the reported sightings of egg-bearing females from the Queensland east coast were from waters where turbidity would be low. However, the non-rare occurrence of egg-bearing females in baited crab pots in estuarine locations is somewhat perplexing. It could be a consequence of the entrapment of mature, stage-V females in pots, but this is unlikely as this should then be a fishery-wide phenomenon. Alternatively, it could be the consequence of appropriate water quality conditions in some inshore locations, as speculated previously [40, 41].

The seasonality of sighted egg-bearing females provided some evidence that the timing of spawning may have a latitudinal gradient related to water temperature [17], occurring later at more southern latitudes and slightly earlier at more northern latitudes (noting the work occurred in the Southern Hemisphere). We suggest that spawning migration occurs when females have advanced ovarian development, which is regulated by hormones, nutrient acquisition and a complex interplay of environmental factors [45]. Whilst a rapid decrease in estuarine salinity has triggered downstream movement of female *S. serrata* in some locations [10], and thus potentially stimulate offshore migration, spawning migration has been observed in other locations when decreases in salinity could not be a trigger [6]. Further research is required to better determine the causes or cues that trigger spawning migration.

### Spawning migration to offshore waters

Extreme fecundity (i.e., millions of eggs per batch) is part of the reproductive strategy of the genus *Scylla* and likely compensates for high larval loss resulting from offshore spawning. Only those larvae that survive and successfully recruit back to inshore estuaries have a chance of effectively contributing to the next generation [18, 19, 45]). Spawning and egg incubation by *Scylla* species is well studied under aquaculture conditions. Females preferentially select fine sand substrates during spawning [46], which involves internal fertilisation, extrusion of initially loose eggs that are gathered into an egg-mass and attached to the setae of the abdominal pleopods. Females actively maintain their egg-mass, removing eggs infected by fungi, polychaetes or nematode worms [16]. Clean water quality is a key requirement of successful mud crab hatcheries [34]. On the basis of this aquaculture research, combined with our field results, we speculate that wild female *S. serrata* seek out locations during their spawning migration with features that support successful egg incubation, so as to maximise successful egg-hatch and larval production. In addition to previously identified optimum temperature and salinity, we suggest that females seek locations with low turbidity and sandy or coral-grit substrates (which often co-occur), thereby minimising the chance and/or rate of eggs becoming infected. In many regions where mud crabs inhabit estuaries, this necessitates an offshore migration. Offshore continental shelf waters generally have more stable temperature and salinity than inshore waters [21]. The temperatures recorded by the microPAT satellite tags (26–28 °C from east coast sites, and 26–30 °C in the Gulf of Carpentaria) are near the optimum embryonic development range recommended for aquaculture hatcheries [34]. While the spawning migration may involve movement against prevailing ocean currents in some locations [10], movement observed in the current study by microPAT tagged females (i.e., release and pop-up location) were both with and against prevailing ocean current, suggesting that the spawning migration may not be universally against prevailing currents.

Previous studies have inferred that egg-bearing female *S. serrata* feed at lower rates than non-egg-bearing individuals and so would not be attracted to and caught in baited crab pots [17]. However, in the current study, egg-bearing females were repeatedly caught in baited pots in certain locations. Speculation that female *S. serrata* have a capital breeding strategy, whereby females rely on stored energy reserves during ovarian development [10] is inconsistent with our field results. Females with varying stage of ovarian development were observed during complimentary field-based research where ovarian development was assessed externally and non-lethally (as per Fig. 2) for approximately 6000 wild, estuary-caught females. Laboratory dissection and macroscopic assessment of ovary development for approximately 1000 of these supported that late stage-V ovary development is visible externally (Robins unpublished). Late inter-moult females with advanced stage-V ovary development (i.e., tertiary vitellogenesis [33], Fig. 2) are expected to spawn within two to three weeks [34]. Our observation was that ovarian development occurs in inshore habitats where food is abundant.

The energy necessary for development of millions of eggs (per egg-mass) and offshore movement has led to speculation that females may use energy-conserving strategies, such as using selective tidal stream transport to move offshore [18, 23, 44]. The reported occurrence of egg-bearing females in baited crab pots raises the possibility that females acquire energy (i.e., feed, see also [47]) during the spawning migration, but it may be that they mostly migrate along depth contours where crab pots are not commonly set. This aspect requires further research. The satellite tagging data provided some evidence that movement by spawning females included active swimming behaviour that was synchronised with the tidal phases, but further verification is required.

The microPAT data, collated sightings and published literature support the paradigm of an offshore spawning migration by female *S. serrata*. However, multiple reliable reports of egg-bearing females in inshore and estuarine environments implies that the spawning migration is spatially variable. Offshore migration may be more prevalent in regions where inshore water quality conditions or prevailing currents are not suitable for larval survival. Coastal and offshore spawning populations occur elsewhere for some species. For example, flounder (*Platichtys flesus*) in the Baltic Sea have genetically distinct offshore and coastal-spawning populations, although they share feeding grounds in coastal waters [48]. In other locations (e.g., near the California Current [49]), variable life history strategies have been hypothesised to improve recruitment of offspring, given the prevailing currents at different times of year, with pelagic phases being a means of achieving movement between oceanic and inshore habitat types rather than a dispersal mechanism. The results of Shanks and Eckert [49] provide an alternative explanation of the importance of widespread larval dispersal to a population’s survival and whether it is a species’ life history or the local environment and oceanography that determines the ‘best’ reproductive strategy. Whilst our results support a predominately offshore spawning strategy for the *S. serrata,* we recommend further research into the spawning migration to better understand locations that provide an effective source of larvae for estuarine recruits. We further note that the larval requirement for specific water quality parameters presents a potential barrier to recruitment, which is relevant to the management of coastal environments, and something which may also be vulnerable to climate events (e.g., marine heat waves).

### Implication for fisheries

Worldwide, there are different regulations regarding the harvest of female mud crabs; with the State of Queensland being one of the few where female harvest is prohibited. Therefore, the population dynamics and mortality of females is relatively natural in this jurisdiction. Hewitt et al. [10] suggested that the spawning migration of female *S. serrata* was terminal, despite the species’ ability to spawn multiple times after a single mating event and is contrary to other reports [6, 17]. The satellite tagging results lead us to concur with Hewitt et al. [10] that most female *S. serrata* likely die during or immediately after their spawning migration, with mortality related to distance moved offshore. In jurisdictions where females are harvested, management should consider the impacts of fishing mortality on inshore mature female biomass as many females are likely to only participate in a single spawning migration. For example, any minimum size limit for female harvest should ensure that a significant proportion of the female population is mature and not exposed to fishing mortality. Where available, mature female biomass should be estimated prior to the onset of the spawning season (e.g. [50]), and fishing mortality be reduced if necessary to ensure sufficient mature females are available to participate in the spawning migration.

## Conclusions

The current study combined information collected on the opportunistic sightings of female *S. serrata* with the novel application of the smallest available pop-up satellite archival tag to gather empirical data on the direction, seasonality, behaviour and movement of spawning females. Combined, the evidence indicates that the offshore spawning migration is variable, depending on local oceanographic and bathymetric conditions. This has consequences for larval distribution and the genetic and demographic connectivity of this widely distributed and important fishery species. Judicious application of pop-up satellite archival tags to brachyuran crabs can provide novel insights into their spawning migration and could be more broadly applied.

## Electronic supplementary material

Below is the link to the electronic supplementary material.

Supplementary Material 1

Supplementary Material 2

## Data Availability

The data collected during the current study are available from the corresponding author (JBR) on reasonable request.
