# Supplementary material for: Offshore movement by the portunid crab Scylla serrata (crustacea: decapoda): new insights into the behaviour and ecology of females migrating to spawn from micro pop-up satellite archival tags
Source: Mov Ecol. 2026 Mar 18;14:29. doi: 10.1186/s40462-026-00636-y (PMC13112637; doi:10.1186/s40462-026-00636-y)
Supplement: Supplementary file 1 — Supplementary Material 1 [file 40462_2026_636_MOESM1_ESM.docx]

Movement Ecology

**Supplementary Material**

**Offshore movement the portunid crab *Scylla serrata* (Crustacea: Decapoda): new insights into the behaviour and ecology of females migrating to spawn from micro pop-up satellite archival tags**.

© The State of Queensland (through the Department of Agriculture and Fisheries) 2025

Nicholas J. Stratford^1^, Samuel M. Seghers^1^, Nicole Flint^2^, & Julie B. Robins^3a^

^1^Department of Agriculture and Fisheries, Northern Fisheries Facility, Cairns Queensland 4870 Australia

^2^CQUniversity, North Rockhampton Queensland 4701 Australia

^3^Department of Agriculture and Fisheries, Ecosciences Precinct, Brisbane Queensland 4102 Australia

^a^ Corresponding author: [julie.robins@daf.qld.gov.au](mailto:julie.robins@daf.qld.gov.au); ORCID 0000-0002-7126-5394; Scopus Affiliation ID: 60028929

**Flyer requesting report of sightings of egg-bearing female giant mud crabs *(Scylla serrata*) distributed directly to fishers and vis social media.**


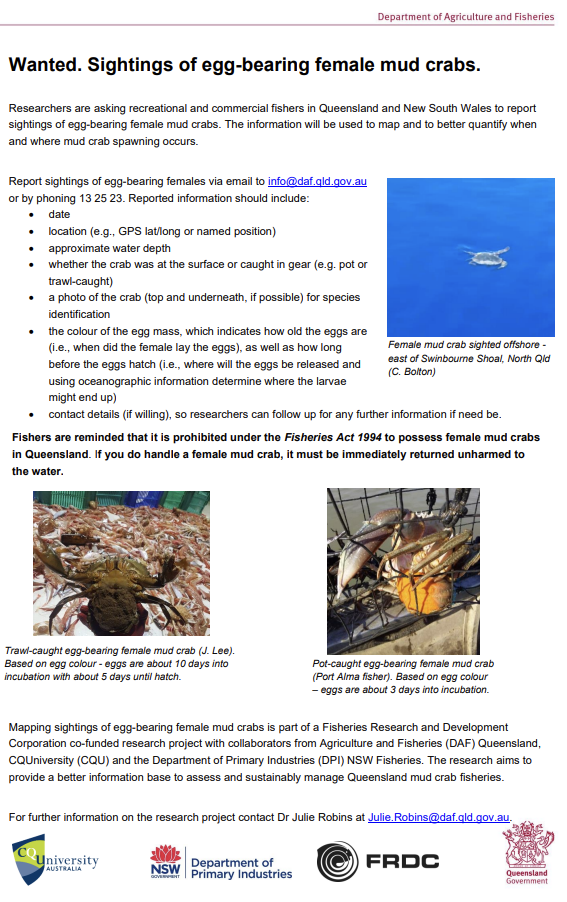


**Table S1** Nominal regions within Queensland waters relevant to giant mud crabs (*Scylla serrata*), their female spawning migration and the Queensland crab fishery, which harvests males >150 mm carapace width. East coast (EC). Gulf of Carpentaria (GoC).

| Region Name | Latitude (°S) | Key landmarks | Commercial logbook grids^a^ |
| --- | --- | --- | --- |
| EC far north | 10.5 to 15.0 | Cape York to Starke River, including Princess Charlotte Bay | B4, B5, B6, C6, C7, C8, D8, C9, D9, D10, D11, E11, F11, D12, E12, F12, G12 |
| EC north | 15.0 to 19.0 | Cooktown to Ingham, including Hinchinbrook Channel | G13, G14, G15 G16, H16, H17, I17, I18, H19, I19, I20 |
| EC north central | 19.0 to 20.5 | Townsville to Bowen, inc. Edgecombe Bay | I21, J21, K21, L21, K22, L22, M22, M23 |
| EC central | 20.0 to 22.5 | Arlie Beach to Yeppoon, including Broadsound | N23, N24, O24, N25, O25, O26, P26, O27, P27, Q27, R27, P28, Q28, R28 |
| EC south central | 23.0 to 24.5 | Fitzroy River to 1770, including The Narrows, Gladstone and Eurimbula Creek | Q29, R29, S29, R30, S30, S31, T30, T31, U31 |
| EC south | 24.5 to 26.5 | Bundaberg to Sunshine Coast including Great Sandy Strait | T32, U32, V32, W32, U33, V33, W33, U34, V34, W34, U35, V35, W35 |
| EC south-east | 26.5 to 28.5 | Sunshine Coast to NSW border | V36, W36, W37, W38, W39, X39 |
| GoC north | 10.5 to 13.0 | Cape York to Weipa | AB8, AB7, A7, AB6, A6, A5 |
| GoC central | 13.0 to 17.0 | Aurukun, Pormpuraaw, including the Mitchell and Nassau Rivers | AC11, AB11, AB10, AC10, AC9, AB9 AB13, AC13, AB12, AC12 AB14, AC14, AD16, AC16, AC15, AB15 |
| GoC south-east | 17.0 to 18.0  140.5 to 141.5°E | Gilbert River to Karumba, including the Norman River | AE18, AD18, AC18, AD17, AC17 |
| GoC west | 16.5 to 18.0  138.0 to 140.5°E | West of Karumba to the NT border | AI16, AH16, AG16, AF16, AG17, AF17, AG18, AF18 |

^a^ https://www.business.qld.gov.au/industries/farms-fishing-forestry/fisheries/commercial/report/logbook/map

**Table S2** Seasonality of reported sightings of female giant mud crabs (*Scylla serrata*) in offshore locations (egg-bearing and non-egg bearing) and in inshore locations (egg-bearing) of Queensland waters by region - east coast (EC) and Gulf of Carpentaria (GoC) between October 2020 and June 2024. Fishery regions defined in Table S1.

| **Fishery region** | **Month** | | | | | | | | | | | |
| --- | --- | --- | --- | --- | --- | --- | --- | --- | --- | --- | --- | --- |
|  | **9** | **10** | **11** | **12** | **1** | **2** | **3** | **4** | **5** | **6** | **7** | **8** |
| EC north | 8 | 6 | 7 | 1 | 3 |  | 1 | 3 | 1 |  | 4 | 2 |
| EC north central | 2 |  |  |  |  |  |  |  |  |  |  |  |
| EC central |  | 1 | 1 | 2 | 1 |  | 1 |  |  |  |  |  |
| EC south central |  | 1 | 1 |  |  |  |  |  |  |  |  |  |
| EC south |  | 1 |  |  | 1 |  |  |  |  |  |  |  |
| EC south-east | 2 | 16 | 8 | 2 | 1 |  | 1 | 2 | 1 | 1 | 2 | 2 |
| GoC north |  | 4 |  |  |  | 1 | 1 |  |  |  |  |  |
| GoC central |  | 1 |  |  |  |  |  | 1 | 2 |  |  |  |
| GoC south-east |  | 2 |  | 1 |  |  |  | 3 |  |  |  |  |
| GoC west |  |  |  |  |  |  |  |  |  |  |  |  |

**Table S3** Water depth (m) where reported female giant mud crabs (*Scylla serrata*) were sighted in offshore locations (egg-bearing and non-egg bearing) and in inshore locations (egg-bearing) of Queensland waters by region - east coast (EC) and Gulf of Carpentaria (GoC) between October 2020 and June 2024. Fishery regions defined in Table S1.

| **Fishery region** | **Depth class (m)** | | | | | |
| --- | --- | --- | --- | --- | --- | --- |
|  | **0-5** | **5-10** | **10-20** | **20-50** | **50-100** | **>100** |
| EC north | 28 | 2 | 1 | 3 | 2 |  |
| EC north central |  | 2 |  |  |  |  |
| EC central | 3 |  | 1 | 1 |  | 1 |
| EC south central | 1 |  |  | 1 |  |  |
| EC south |  | 1 |  | 2 |  |  |
| EC south-east | 1 | 15 | 14 | 7 |  | 1 |
| GoC north |  |  | 2 | 4 |  |  |
| GoC central | 4 |  |  |  |  |  |
| GoC south-east | 6 |  |  |  |  |  |
| GoC west |  |  |  |  |  |  |

**Detailed information from individual microPATs, see Table 1 of main text for details.**

**Karumba, Gulf of Carpentaria, deployed October 2023**

MicroPATs were deployed on female giant mud crabs in the south-east Gulf of Carpentaria (GoC) in early October 2023 (i.e., austral spring, end of the dry season), when water temperatures increase and build-up conditions of the monsoon season start, but rain is unlikely (BOM 2019). October coincides with the reduced catches of legal males in the commercial sector in this region and anecdotally when mature female giant mud crabs move away from estuaries and the coastal flats to ‘offshore’ locations. Of the five microPATs deployed in the GoC, none were retrieved, so all results for this region are based on summary data from the four tags that transmitted to Argos satellite network while floating after release.

*PTT ID 253090, 160 mm CW*

After the programmed duration of 60 days, microPAT 253090 released at a depth of 19 m approximately 103 km straight-line distance north-northwest of its release location. Depth summary data (min/max) suggests that crab-253090 remained within the Norman River estuary (at ~10 m depth) until about day-15 post-release, then moved to deeper offshore water (Fig. S1). Crab-253090 was active for next 19 days, moving between depths of 0 m (i.e., animal at surface), to a maximum of 43 m, then decreasing to 20 m depth. On day-36 post-release, crab-253090 remains consistently at a depth of around 20 m (± 2 m i.e., tidal range see Fig. S1), suggestive of offshore benthic behaviour, possible sedentary. Temperature-at-depth (from the LightLoc file) indicates initial water temperature of ~27.5°C, which increases over the 60 days to ~30°C. We infer that crab-253090 continued to move, possibly searching for an appropriate conditions to incubate its eggs. Following discussions with local fishers and examination of navigation charts (noting the Gulf of Carpentaria is poorly surveyed), we suspect the crab may have travelled towards offshore areas via an old river channel, reaching a maximum depth of 43 m. After 35 days post-release, the temperature remained at stable (~30°C). The depth data then follows the diurnal tide cycle, suggesting the crab was sedentary (Fig. S1). At 29 to 30°C, incubation of giant mud crab eggs is estimated to take 11 to 10 days (Heasman and Fielder 1983). If so, crab-253090 was sedentary at-depth, either recovering from egg-incubation or had extruded and was incubating another batch of eggs. Giant mud crabs are able to produce up to three batches of egg per inter-moult (Quintello and Parado-Estepa 2003). Sediment type at the pop-off location is unconfirmed, but anecdotal reports suggests it is likely coarse, clean sand. Given the pop-off location (16.61947 °S, 140.43152 °E), and lack of change in the depth profile for the final 25 days of the tag duration, there is no evidence (in the depth or temperature data) that crab-250390 returned to shallow inshore or estuarine waters.

*PTT ID 253091, 169 mm CW*

After the programmed duration of 60 days, microPAT 253091 popped-up from a depth of 21 m approximately 106 km straight-line distance north-northwest of its deployment location. Depth summary data (min/max) suggests that crab-253091 remained in the estuary for 9 days post-release before moving to deeper offshore waters. Large changes in the min/max depth indicates crab-253091 was constantly moving, likely in an offshore direction as water depth gets deeper, before stabilising at day-17 post-release in about 22 m water depth. Crab-253091 remains consistently at this depth for the remainder of the tag deployment. The recorded depth data closely follows the diurnal tide cycle (Fig. S1). Temperature-at-depth indicates initial temperature of ~25.8°C, which increases to ~30°C at 58 days post-release. Given the pop-off location (16.57403°S, 140.50987°E) and lack of movement for the final 43 days post-release, there is no evidence that crab-250391 returned to shallow inshore or estuarine waters. Whether crab-253091 spawned once or more is unknown.

PTT ID 253092, 166 mm CW

This tag failed to connect to the Argos network and no data were recovered.

PTT ID 253093, 178 mm CW

After 41 days, microPAT 253093 prematurely released at a depth of 19 m before washing ashore about 54 km straight-line distance north of the Norman River. The 20 m depth contour is approximately 30 km offshore in the vicinity of where the tag washed ashore. Depth summary (min/max) data suggests that crab-253093 remained in the estuary or inshore waters for 28 days post-release before moving to deeper offshore waters. Crab-253093 was somewhat settled at a water depth of 20 m for 5 days before the tag prematurely detached from the crab. Temperature-at-depth indicates initial temperature of ~26.8°C, which increased to 28.8°C at ~ 29 days post-release (Fig. S2). Although data collection occurred for only 38 days, crab-25093 showed similar patterns in its depth profile (Fig. S2), moving to similar depths and water temperatures as the other crabs. Visual analysis of tag drift patterns and currents in the south-east Gulf of Carpentaria at the time suggest that microPAT 253093 is likely to have detached from the crab near where the other tags popped up, then drifted south-east for 22 days, when at 60 days post-release and positioned at 17.00323°S, 140.95843°E, the tag transmitted data to the Argos satellite network as programmed. The summary light data is inadequate to confirm the cause of tag loss (e.g., predation). It is unknown whether crab-253093 had spawned and had settled on the bottom to incubate an egg-mass or a mishap occurred to the crab or the tag, resulting in the tag’s premature detachment.

*PTT ID 253096, 167 mm CW*

After the programmed duration of 60 days, microPAT 253096 released at a depth of 19 m approximately 73 km straight-line distance north-northwest of its release location (-16.95468°S, 140.38788°E). Depth summary data (min/max) suggests that crab-253096 remained in the estuary for 14 days post-release before moving to deeper offshore waters (Fig. S2). Depth summary data suggest crab-253096 moved for a further 14 days, with maximum depth gradually increasing to and stabilising at 20 m, at day 29 post-release. The depth summary data indicate minor activity at day 41 post-release. The recorded depth data closely follows the diurnal tide cycle. The first temperature-at-depth data was 28.0°C on day 17 post-release. Temperature-at-depth fluctuated slightly (27.8 to 29.4°C), eventually reaching ~30°C at 59 days post-release.


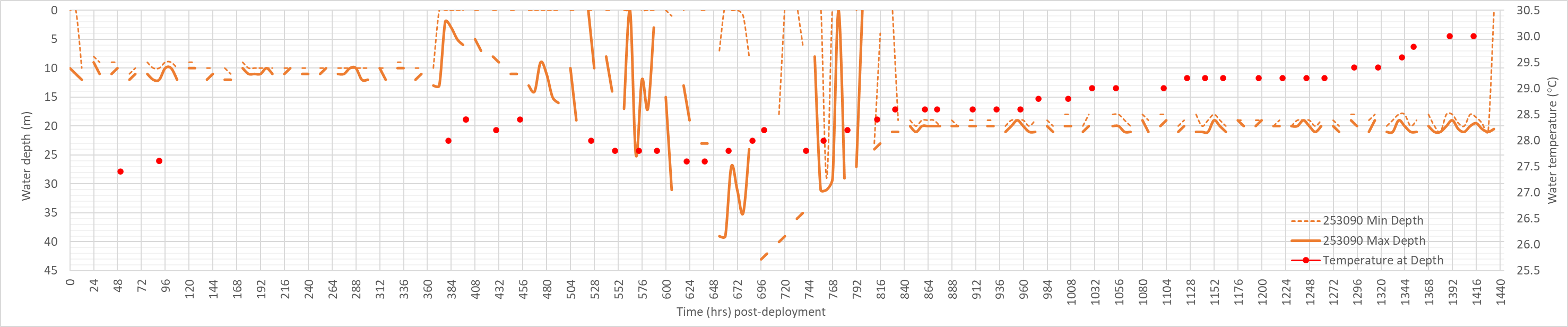


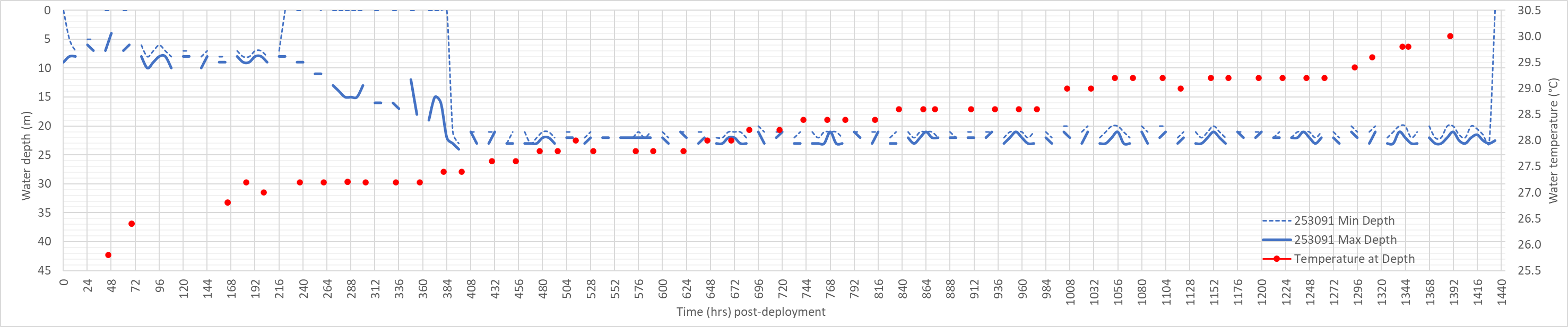


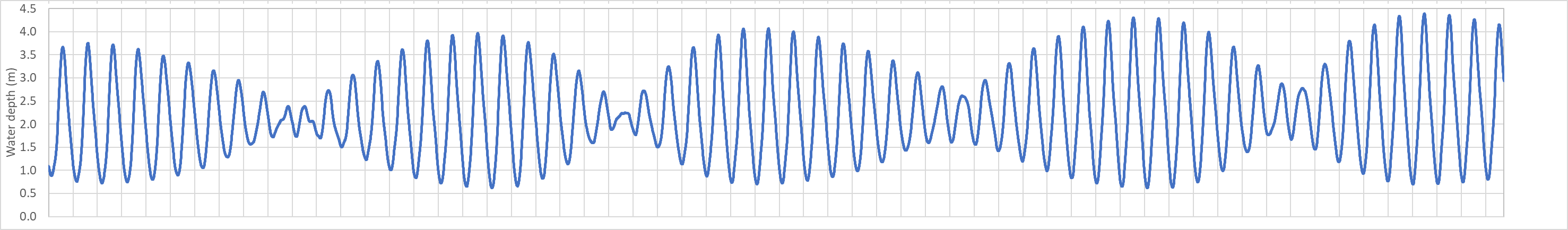


*Fig. S1. Depth and temperature profiles of summary data transmitted to Argos from microPATs attached to female giant mud crabs in the Gulf of Carpentaria south-east region (see Table 1 of main text for details). Lower panel shows observed tide Karumba (station number 071004A).*


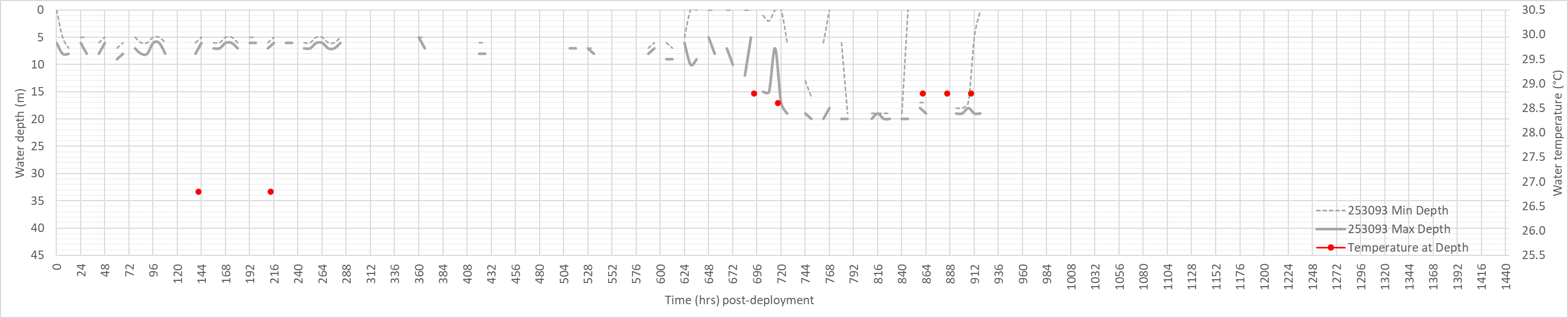


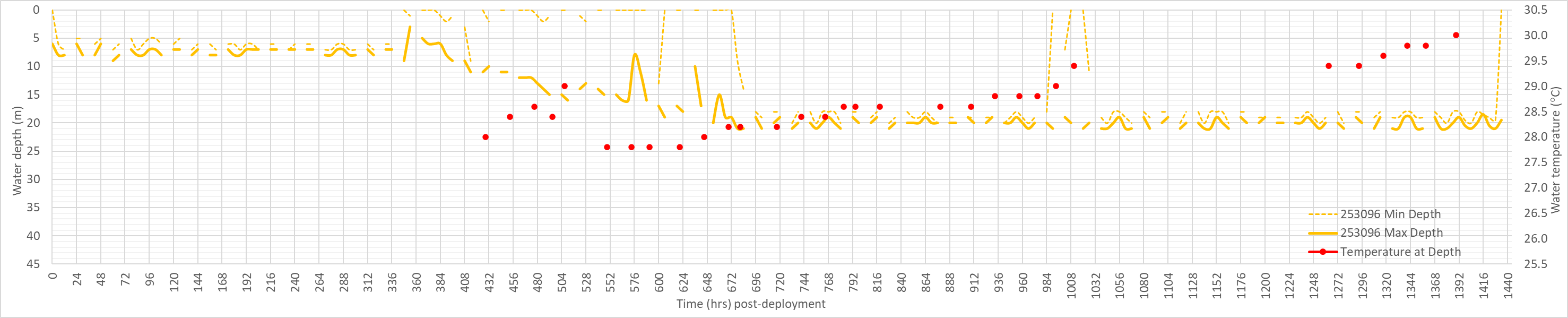


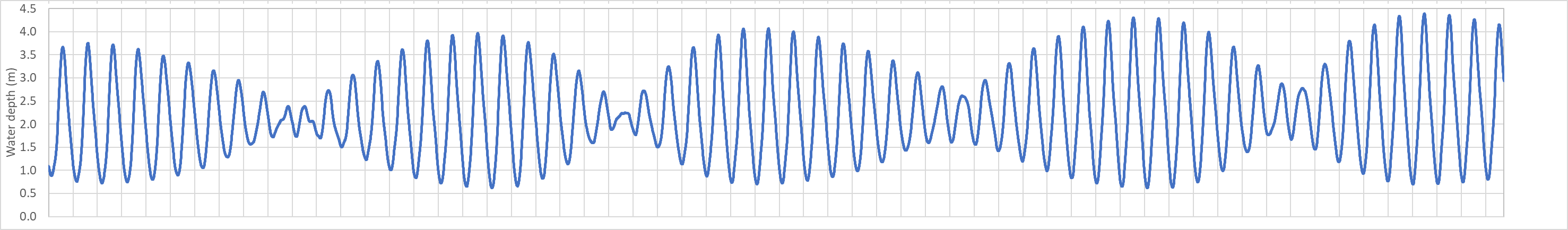


*Fig. S2. Depth and temperature profiles of summary data transmitted to Argos from microPATs attached to female giant mud crabs in the Gulf of Carpentaria south-east region (see Table 1 of main text for details). Lower panel shows observed tide Karumba (station number 071004A).*

***Hinchinbrook Island (Missionary Bay), Queensland east coast, deployed October 2023***

Female giant mud crabs were tagged at Missionary Bay, Hinchinbrook Island, in mid-October when water temperatures were increasing, and increased sightings of egg-bearing females were reported by commercial fishers in the region, commonly referred to as the ‘Wet Tropics’. October is prior to the onset of the monsoon season, and main months of rainfall and flooding (i.e., January to April, BOM 2019). Anecdotally, commercial fishers report mature females are in higher densities on the coastal flats adjacent to mangrove lined estuaries. Two of the Queensland northeast coast microPATs were retrieved and allowed contrast between summary data transmitted to Argos and archived data recorded at 30 second intervals.

*PTT ID 253088, 150 mm CW – egg-bearing*

After the programmed duration of 30 days, microPAT 253088 released from a depth of 20 m, about 11 km straight line distance from its release location (-18.15738°S, 146.29133°E). This microPAT was recovered from the beach of North East Bay, Palm Island, 74 km southeast of its pop-up location. Based on depth summary data (min/max), this egg-bearing female moved to deeper water within four days post-release (fig. S3), and remained at this depth, indicative of offshore benthic behaviour, until the programmed pin-burn and the tag popped-up. There is no evidence in the depth or temperature summary data that crab-253088 returned to shallow or estuarine waters. At water temperatures between 25 and 30°C, egg-incubation is estimated to take 10 to 15 days (Heasman and Fielder 1983; Quintello and Parado-Estepa 2003). The egg mass of crab-253088 was already about three days post-spawn based on visible characteristics (i.e., bright orange colour, lack of black pigmentation associated with more advanced egg development) and should have hatched at most 12-days post-release. Temperature-at-depth indicates an initial water temperature of ~26°C, gradually increasing to ~27°C at 20 days post-release (Fig S3). These values concur with 30-second archive data, indicating that the summary data transmitted to the Argos satellite network captured key changes in the depth and temperature encountered in the current study by female giant mud crabs during their spawning migration. Based on inspection of the 30 second archive data (Fig S3), we infer that crab-253088 immediately left the shallow coastal flat when it was released, and moved into deeper, cooler water. We aligned the depth data with the nearest observed 10 minute tide records (Cardwell, station number 035012A) to examine evidence of location in the water column (i.e., surface or sea floor), and tidal water movement (i.e., tidal stream transport). On the flood tide, crab-253088 moved up and down within the water column moving closer to the surface during the flood tide and closer to the sea floor during the ebb tide. After about three days, crab-253088 had a stable depth profile, which continued until the microPAT detaches from the crab at 30 days post-release. The constant depth profile (~20 m) suggests crab-253088 did not move back into shallow estuarine waters. There is no indication as to whether this crab spawned another egg mass, remained offshore to feed and gain resources to spawn another egg-mass, or died at the site. However, post-spawn female giant mud crabs are resource depleted and actively feed in aquaculture conditions, presumably to acquire resources that would enable further spawning (D. Mann pers. comm 2024).


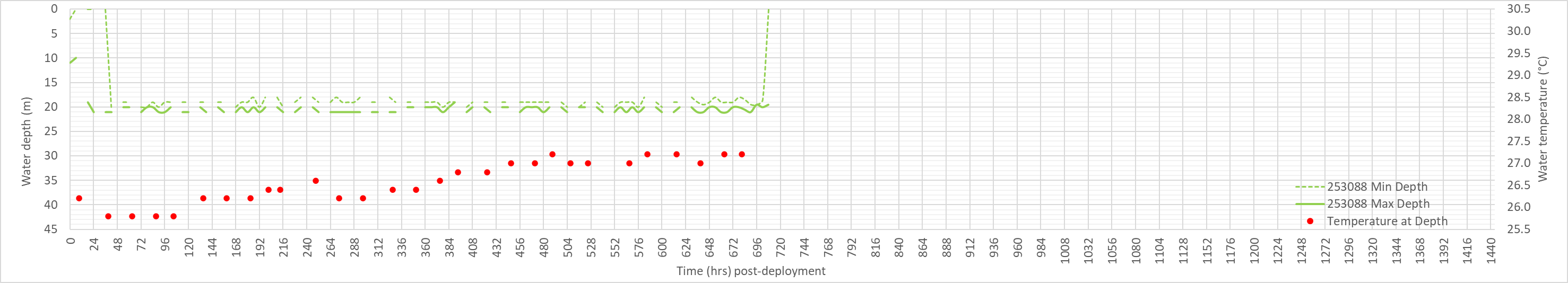


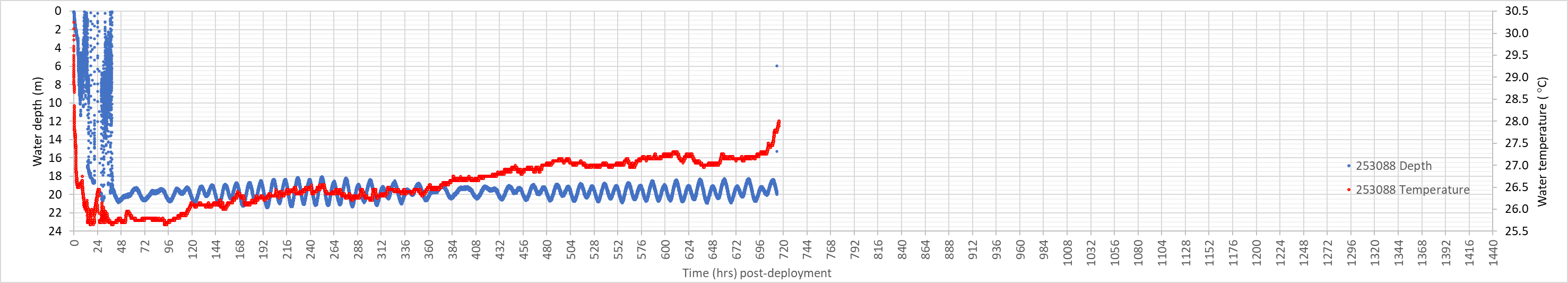


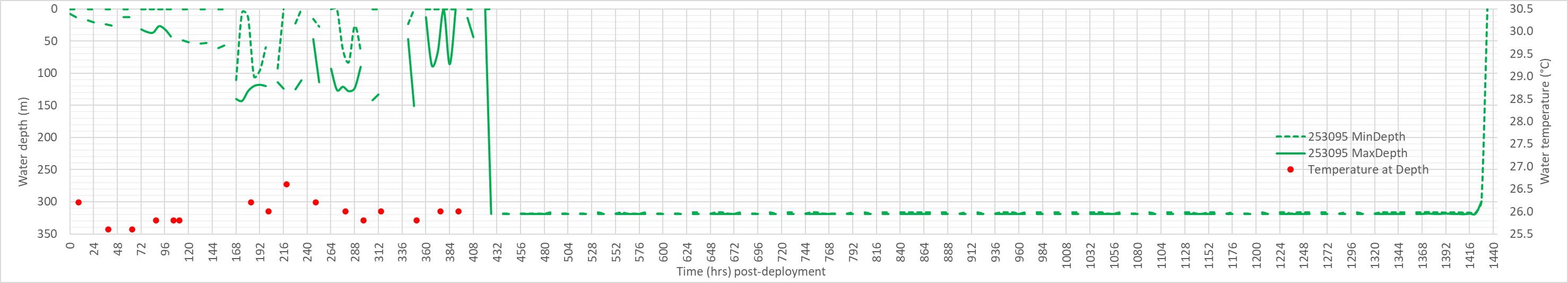


*Fig. S3. Depth and temperature profiles of summary data transmitted to Argos from microPATs attached to female giant mud crabs along the Queensland north-east coast. (see Table 1 of main text for details).*


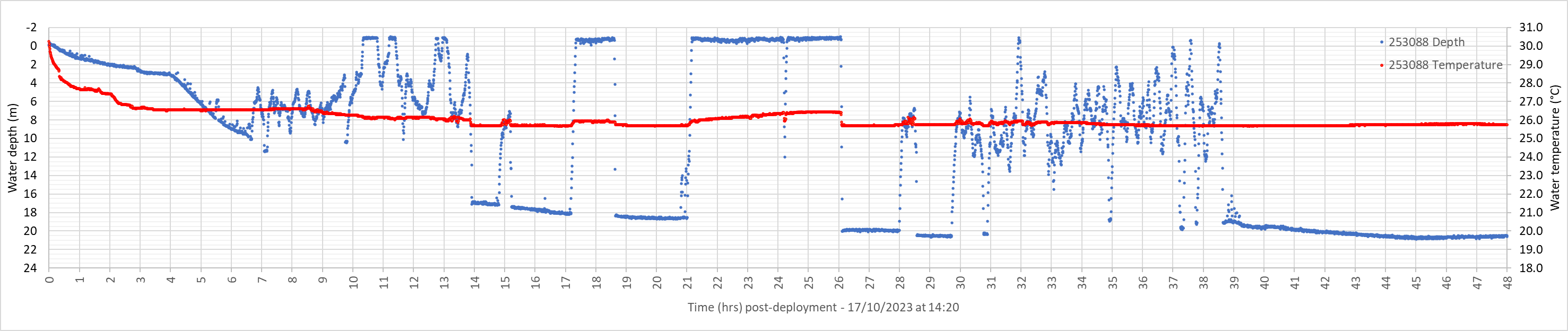


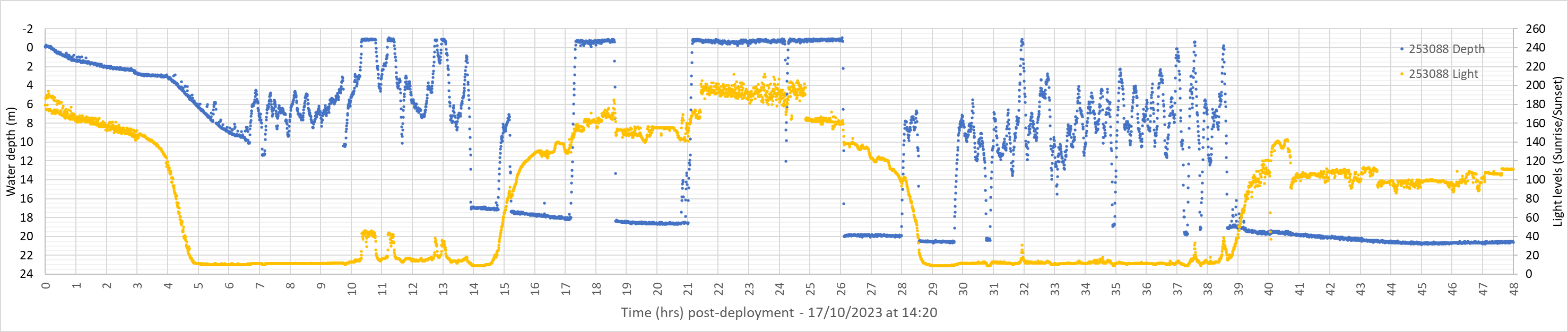


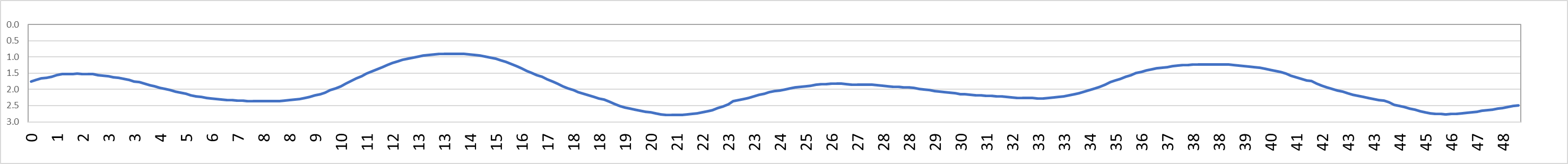


*Fig. S4. Archive 30 second interval data for crab-253088, a 150 mm carapace width egg-bearing female giant mud crab for the first 48hrs post-deployment. (a) depth and temperature), (b) depth and light, (c) observed tide at Cardwell, station number 035012A*.

*
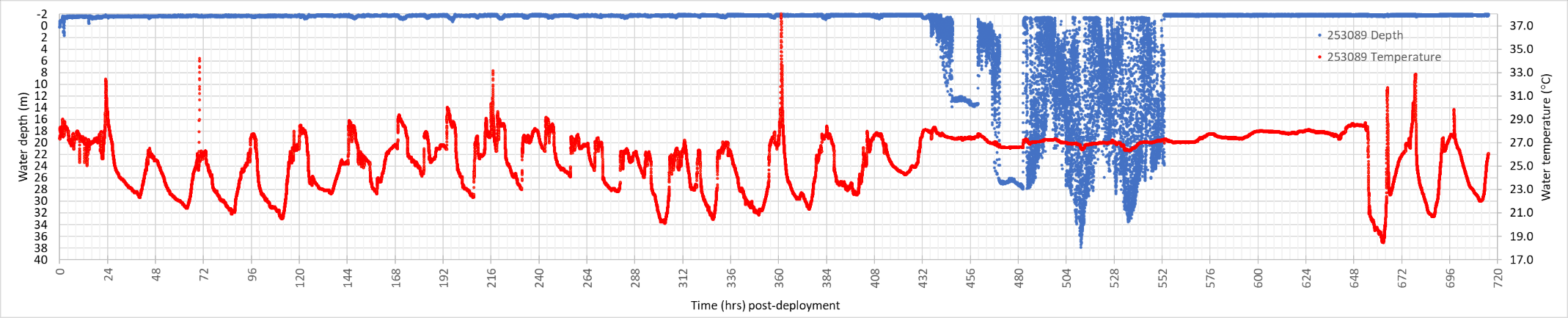
*


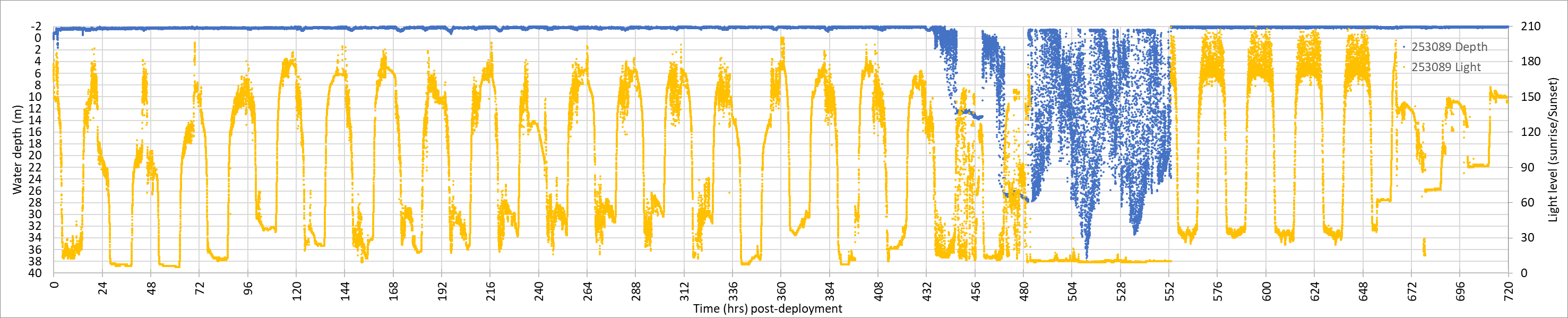


*Fig. S5. Archive 30 second interval data for crab-253089, a 157 mm carapace width female giant mud crab, showing (a) depth and temperature and (b) depth and light level. Likely period of predation indicated by no day/night cycle in the light profile (yellow line) between ~480 and 522 hours post-deployment.*

*PTT ID 253089, 157 mm CW*

MicroPAT 253089 was recovered before its programmed 60 day release date, so no data were transmitted to Argos. This tag was recovered from a beach approximately 39 km northwest of its release location. Archived 30 second interval data was downloaded from the retrieved tag (Fig. S5) from which the following inferences have been made. The depth, temperature and light data suggest that crab-253089 remained in shallow water, likely the coastal flats where it was initially tagged, for about 18 days post-release. The temperature and light data suggest that crab-253089 then moved into deeper water (26-28 m). The light data, which had previously tracked the sunrise/sunset cycle until 20 days post release, showed a 72 hour period of darkness (i.e., light level below 20), during which it is likely the tag had been ingested (Fig. S5). Temperature readings stabilise during this 72 hrs, while the depth data is extremely variable. After 23 days post-deployment, the light data had a cycling pattern indicative of the sunrise/sunset cycle, with the microPAT likely having passed through the predator. Recovered microPAT 253089 was missing its antenna and covered in teeth marks, supporting the inference of predation.

*PTT ID 253094, 147 mm CW*

This tag failed to connect to the Argos network and no data were recovered.

*PTT ID 253095, 168 mm CW*

After the programmed duration of 60 days, microPAT 253089 popped-up from a depth of 320 m, approximately 363 km straight-line distance southeast from its release location. The tag first surfaced outside the Great Barrier Reef, in deep waters north-northeast of Whitsunday Island. Similar to crab-253088, this female moved to deeper, offshore waters within one day post-release. Depth summary data (min/max) suggests that crab-253095 reached deep water (>100 m, indicative of the edge of the continental shelf in the GBR region) by day-7 post-release (Fig. S3). Significant daily differences between minimum and maximum depth (~0 to 150 m) suggests continuous activity by crab-253089, possibly southward movement, which is the main current direction at that time. By day-18 post-release, the depth data indicate crab-253095 had moved into water that was approximately 320 m deep, where it remained until the pin burned at 60 days post-release and the tag popped up. Recorded water temperature at this depth was ~13°C (from the Series Range file). Incubation is unlikely to have been successful at this temperature (Heasman and Fielder 1983), and we assume that this crab had died. If crab-253089 was alive, the prevailing currents and the distance from shore (130 km) suggest it would be implausible for crab-253095 to return to shore. Temperature-at-depth data indicates initial water temperature of ~26°C (Fig S3) and then fluctuates slightly. The last water temperature data in the LightLoc file is at 16 days post-release.

*PTT ID 253097, 156 mm CW*

This tag failed to connect to the Argos network and no data were recovered.

***The Narrows, Queensland central east coast, deployed April 2024***

Female crabs collected from Deception Creek, at the northern end of ‘The Narrows’ (north of Gladstone, central Queensland) were tagged in mid-April 2024, when water temperatures were decreasing, and sightings of mature females were increasing. Release settings on these two microPATs were different to tags deployed in October 2023. These microPATs were programmed such that the tag would commence the release sequence when: (i) the tag was more than 25% dry or shallower than 1 m, or (ii) the tag was at a constant depth ±4 m for longer than 120 hours. These settings were trialled to overcome the loss of data from microPATs that released early, but that never successfully transmitted to Argos satellite network. Early release tags drifted before programmed transmission occurred, likely becoming entangled in estuarine mangrove habitat which interferes with transmission. The release-pin on these microPATs was set to burn at 45 days post-release.

*PTT ID 262949, 166 mm CW*

MicroPAT-262949 popped up after 6 days post-release. Crab-262949 had moved 20 km straight-line distance northwest from Deception Creek to a mangrove island at the mouth of the Fitzroy River. Crab-262949 remained there for several days in amongst mangroves in water less than 0.5m deep, which resulted in programmed ‘mortality’ conditions being met and a premature tag release. A few signals were received by the Argos satellite network (mostly location), but no useful summary data were received.

*PTT ID 262950, 163 mm CW*

MicroPAT-262950 transmitted data to the Argos network at 21 days post-release. Depth summary data (min/max) suggests that the tag had detached three days prior from a depth of 69 m near Henderson Reef, east of Whitsunday Island - approximately 389 km straight-line distance north-northwest of its release location. Crab-262950 remained inshore (≤10 m depth) for 5 days post-release, then moved into deeper water (40-50 m, Fig. S6). The summary data indicate crab-262950 was constantly moving, with depth fluctuating between the surface and depth (presumably near the seafloor), but never settled at a steady depth like some of the other tagged crabs. There is no indication in the light data that this crab was predated. Satellite SST imagery for the region during tag deployment, which includes current vectors, shows a current flowing from south to north up the Capricorn Channel, potentially aiding movement (https://oceancurrent.aodn.org.au/product.php). Once microPAT-262950 was transmitting data to the Argos satellite network, it drifted around but remained close to the pop-up location for 7 days. The lack of substantial movement of the tag (due to tide, current or wind drift) suggests that active swimming and depth choice by crab-262950 is primarily responsible for its large-scale movement (i.e., 389 km in less than 21 days). Data returned from microPAT-262950 indicated it detached from the crab because of the WetDry function. As the crab didn’t appear to stop moving and settle at a depth, it is possible that spawning had not occurred.


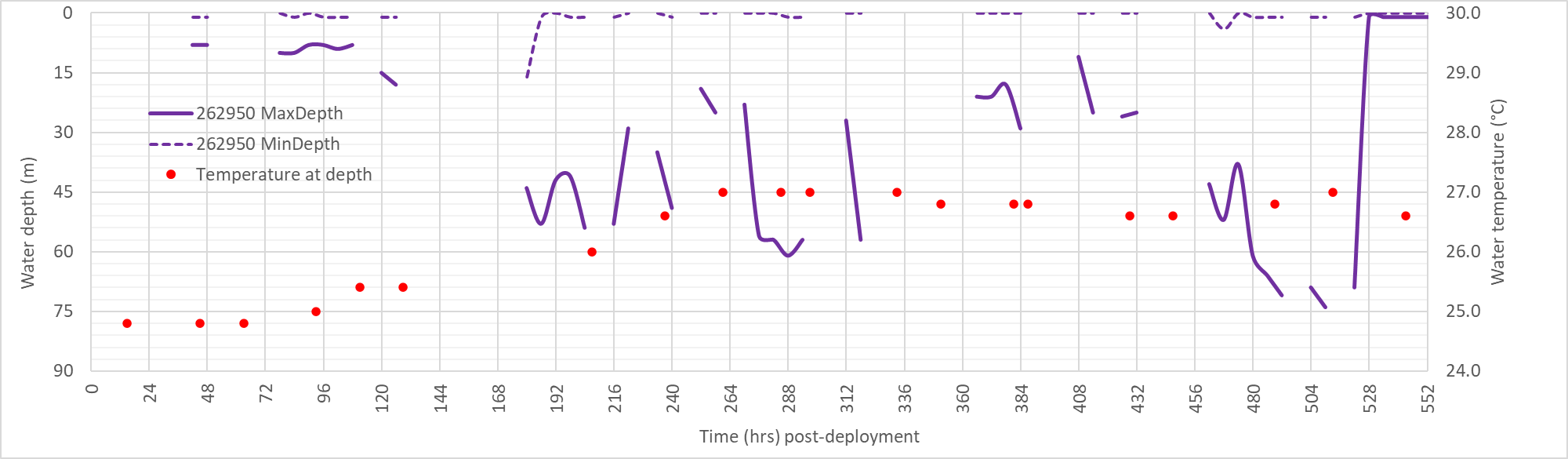


*Figure S6. Depth and temperature profile of summary data transmitted to Argos from microPAT-262950 attached to a female giant mud crab at The Narrows, Queensland east coast*

**Post-processing of microPAT data to estimate geolocation**

Post-processing of tag data using GPE3 statistical modelling (i.e., state-space hidden Markov model accessible in the Wildlife Computer data portal – see <https://static.wildlifecomputers.com/Location-Processing-UserGuide.pdf>) can estimate movement as a time-discrete gridded probability surface (0.25 degree of latitude and longitude, representing approximately 725 km^2^). Location is estimated from PSAT data of light-levels (to estimate twilight i.e., sunrise and sunset), sea surface temperature and bathymetry, being more robust for near-surface readings in clear water (i.e., oceanic conditions). Turbid water, cloud cover, deep diving behaviour, and equinox issues (which change light level reading) make location estimates problematic. We attempted geoprocessing on the PSAT data received or recovered. Inputs to GPE3 modelling include seed locations (time and lat/long at deployment, end and any known locations during deployment) and animal swimming speed (m sec^-1^). Geolocation processing results are provided as extra information in the Supplementary Material as the best fits (based on Score and plausibility of movement). Movement tracks estimated from the GPE3 location processing had variable ‘fit’ (i.e., based on maximum likelihood ‘score’ (0 to 100)), with several of the PSAT data sets unable to have a track estimated due to lack of model convergence or estimated movement track we considered unlikely. Plausible tracks are presented below.

**Karumba crabs**

*PTT ID 253090, 160 mm CW*

Maximum likelihood tracks of estimated movement of the crab fitted with microPAT-253090 had moderate model fit scores for animals speeds of 0.5 to 1.5 m sec^-1^ (Fig. S7). The model could not be fit with an animal speed of 0.1 m sec-1 and could not fit with the precise release location (Table 1 of main text) as it considered these coordinates to be land based and out of bounds.


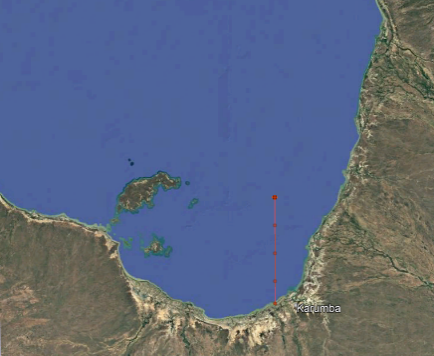

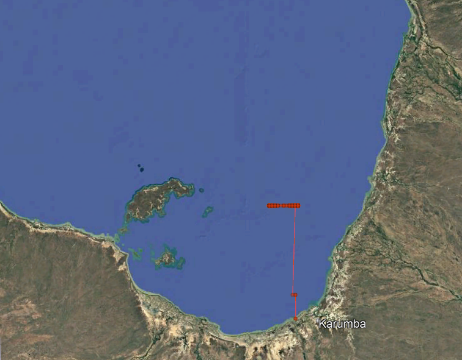

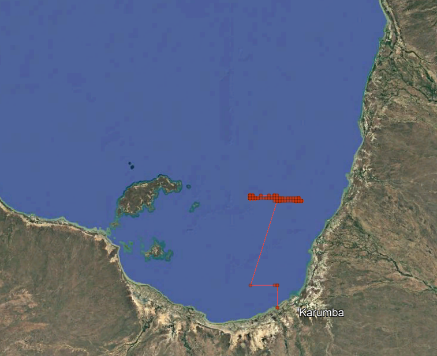

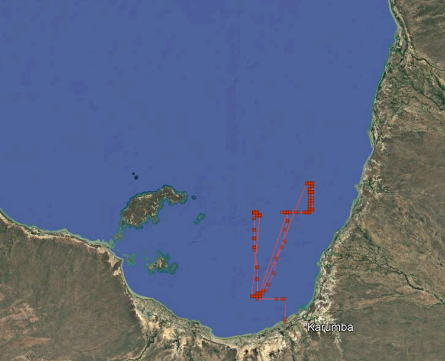

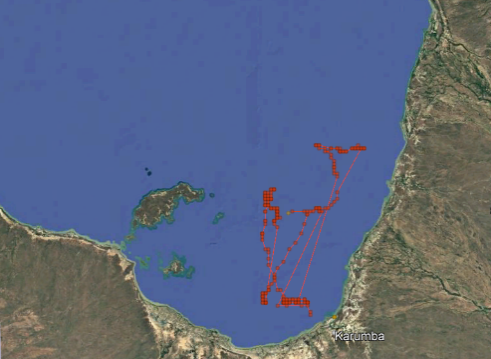


0.85 m sec^-1^ (57.41)

1.5 m sec^-1^ (57.37)

1.0 m sec^-1^ (57.71)

0.75 m sec^-1^ (56.44)

0.5 m sec^-1^ (55.34)

*Figure S7. Maximium likelihood tracks and locations dervied from GPE3 statistical processing for microPAT-253090, for different animal speeds. Model score (goodness of fit 0 to 100) is provided in brackets*.

*PTT ID 253091, 169 mm CW*

Maximum likelihood tracks of estimated movement of the crab fitted with microPAT-253091 had poor model fit scores for all animals speeds tested (i.e., 0.25 to 1.5 m sec^-1^) but did produce tracks of varying plausibility (Fig. S7). The model could not be fit with an animal speed of 0.1 m sec^-1^ and could not fit with the precise release location (Table 1 of main text) as it considered these coordinates to be land based and out of bounds.


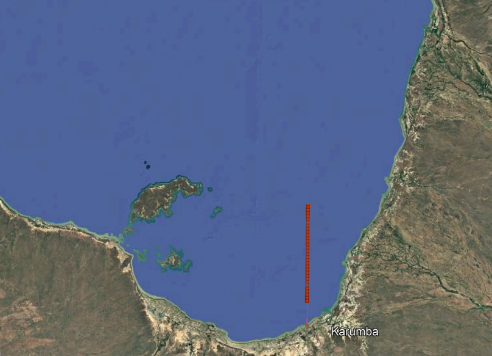

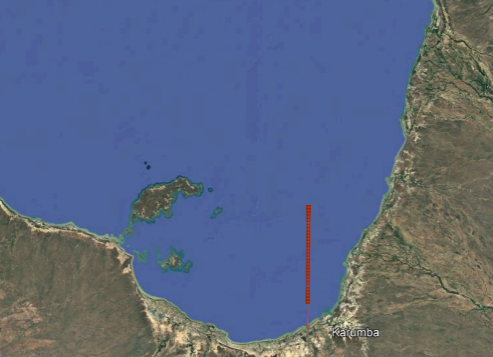

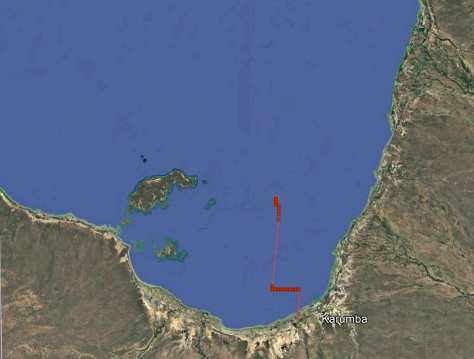

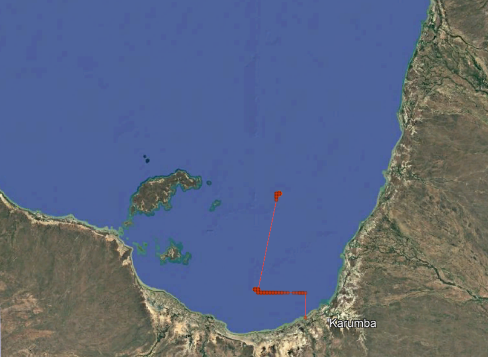

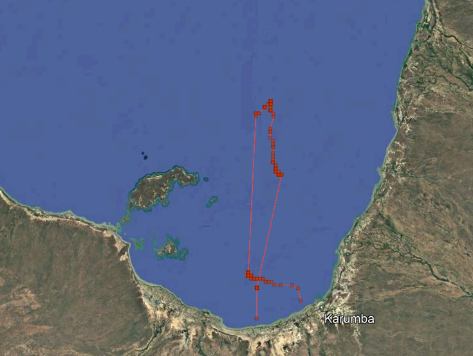


1.5 m sec^-1^ (0.37)

0.5 m sec^-1^ (0.54)

1.0 m sec^-1^ (0.41)

0.75 m sec^-1^ (0.46)

0.25 m sec^-1^ (0.59)

*Figure S8. Maximium likelihood tracks and locations dervied from GPE3 statistical processing for microPAT-253091, for different animal speeds. Model score (goodness of fit 0 to 100) is provided in brackets*.

*PTT ID 253093, 178 mm CW*

Maximum likelihood tracks of estimated movement of the crab fitted with microPAT-253091 had moderate model scores, but we considered them to poorly represent the likely movement track of the animal, with the maximum likelihood fit not encompassing the 20m depth contour line, which the transmitted data indiated that crab had travelled to. The time lapse and confidence intervals of the estimated track is provided in Figure S9 to illustrate the model precision. The model could not be fit with an animal speed of 0.1 m sec^-1^ and could not fit with the precise release location as it considered these coordinates to be land based and out of bounds.


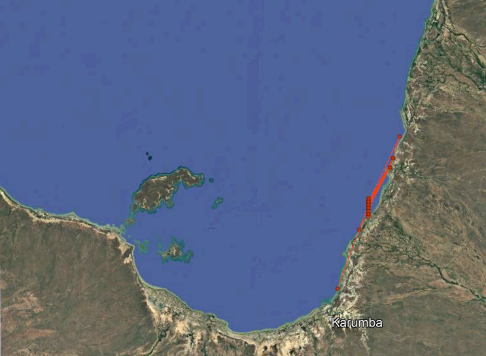

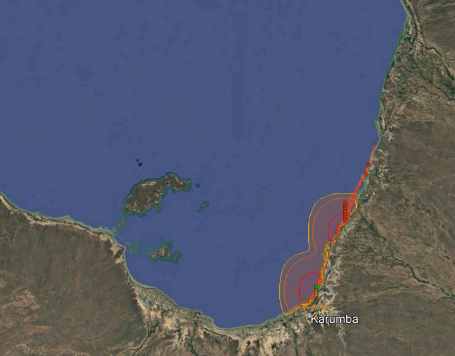


0.75 m sec^-1^ (42.75)

0.5 m sec^-1^ (42.43)

*Figure S9. Maximium likelihood tracks and locations dervied from GPE3 statistical processing for microPAT-253093, for different animal speeds. Model score (goodness of fit 0 to 100) is provided in brackets, with time lapse confidence intervals provided for the 0.75 m sec^-1^ model run*.

*PTT ID 253096, 167 mm CW*

Maximum likelihood tracks of estimated movement of the crab fitted with microPAT-253096 had moderate model scores, and varying degrees of complexity in movement and plausibility (Figure S10). The model could not be fit with an animal speed of 0.1 m sec^-1^ and could not fit with the precise release location as it considered these coordinates to be land based and out of bounds.


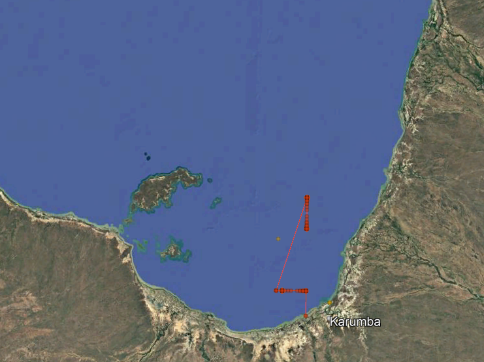

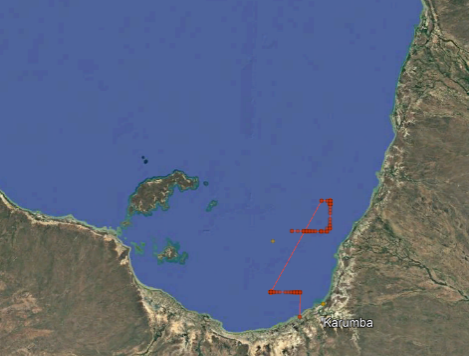

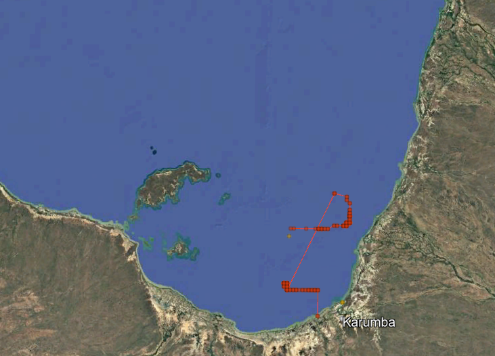

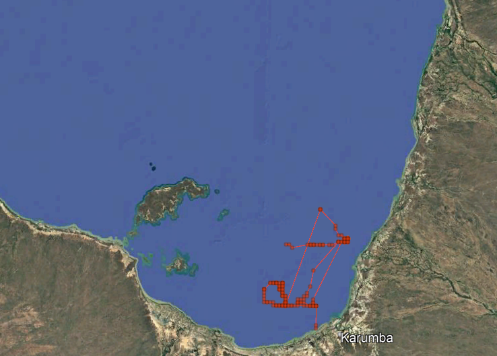

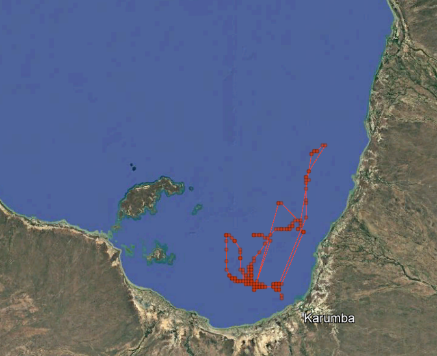


1.5 m sec^-1^ (53.75)

1.0 m sec^-1^ (53.00)

0.85 m sec^-1^ (52.57)

0.75 m sec^-1^ (51.68)

0.5 m sec^-1^ (50.29)

*Figure S10. Maximium likelihood tracks and locations dervied from GPE3 statistical processing for microPAT-253096, for different animal speeds. Model score (goodness of fit 0 to 100) is provided in brackets*.

**Hinchinbrook crabs**

*PTT ID 253088, 150 mm CW – egg-bearing*

No model was able to be fitted to the microPAT data for this crab, as the release location caused a model error (coordinates on land), and even with slight seaward adjustment of release location the model still failed to fit.

*PTT ID 253089, 157 mm CW*

No model was able to be fitted to the microPAT data for this crab, as the release location caused a model error (coordinates on land), and even with slight seaward adjustment of release location the model still failed to fit.

*PTT ID 253095, 168 mm CW*

Maximum likelihood tracks of estimated movement of the crab fitted with microPAT-253095 had moderate model scores, and varying degrees of complexity in movement and plausibility (Figure S10). The model could not be fit with an animal speed of 0.5 m sec^-1^ and could not fit with the precise release location (Table 1 of main text) as it considered these coordinates to be land based and out of bounds. Whilst the initial track (i.e., within the shelf of the Great Barrier Reef), is plausible, we consider that the track east of the outer reef is unlikely.

1.25 m sec^-1^ (60.96)

0.85 m sec^-1^ (59.95)


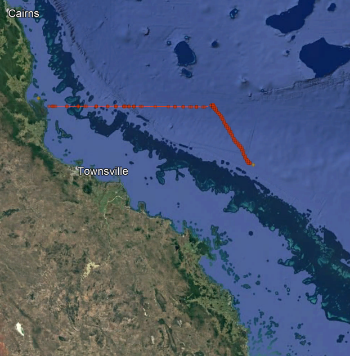

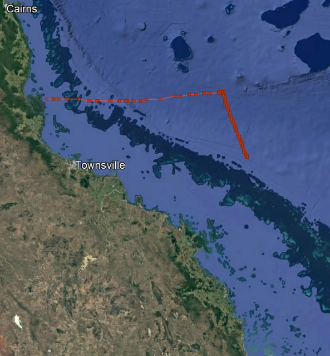

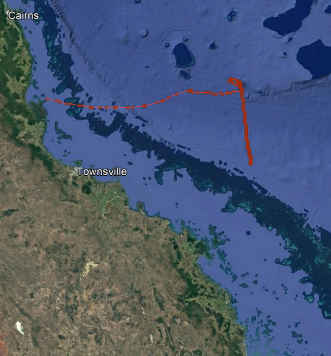

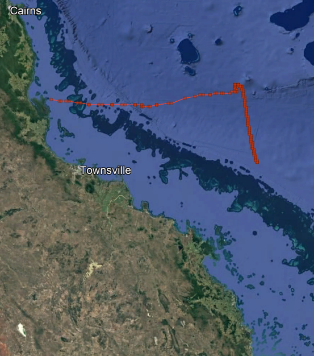


1.5 m sec^-1^ (60.55)

1.0 m sec^-1^ (60.88)

*Figure S11. Maximium likelihood tracks and locations dervied from GPE3 statistical processing for microPAT-253095, for different animal speeds. Model score (goodness of fit 0-100) is provided in brackets*.

**The Narrows crabs**

*PTT ID 262949, 166 mm CW*

No model was able to be fitted to the microPAT data for this crab, as the release location caused a model error (coordinates on land), and even with slight seaward adjustment of release location the model still failed to fit.

*PTT ID 262950, 163 mm CW*

Maximum likelihood tracks of estimated movement of the crab fitted with microPAT-262950 had moderate model scores, and varying degrees of complexity in movement and plausibility (Figure S12). The model could not fit with the precise release location (Table 1 of main text) as it considered these coordinates to be land based and out of bounds. However, we consider the GPE3 estimates of movement tracks for microPAT-262950 the most plausible of all the crabs analysed.

0.75 m sec^-1^ (59.79)

2.0 m sec^-1^ (57.58)

1.5 m sec^-1^ (59.54)


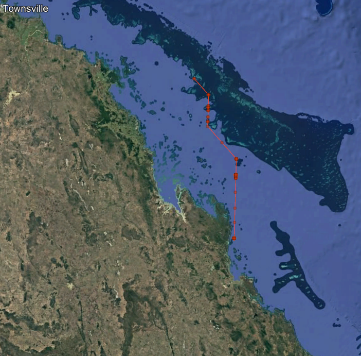

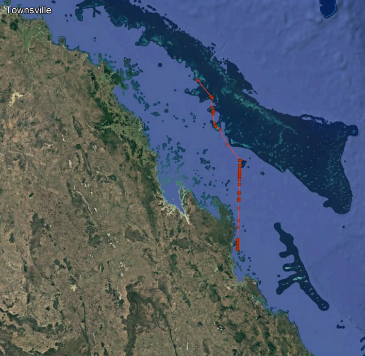

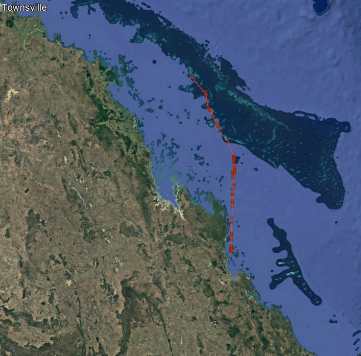

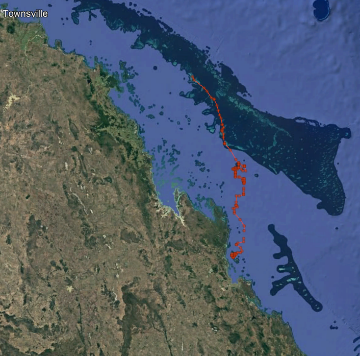

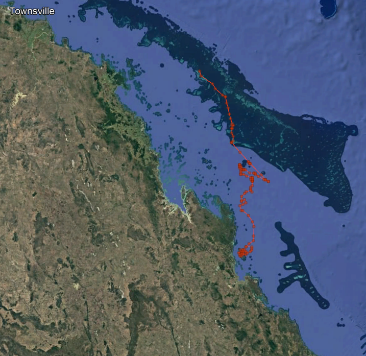


1.0 m sec^-1^ (60.53)

0.5 m sec^-1^ (58.21)

*Figure S12. Maximium likelihood tracks and locations dervied from GPE3 statistical processing for microPAT-2629505, for different animal speeds. Model score (goodness of fit 0 to 100) provided in brackets*.
